# Exploring the Evolutionary History and Phylodynamics of Human Immunodeficiency Virus Type 1 Outbreak From Unnao, India Using Phylogenetic Approach

**DOI:** 10.3389/fmicb.2022.848250

**Published:** 2022-05-18

**Authors:** Ajit Patil, Sandip Patil, Amrita Rao, Sharda Gadhe, Swarali Kurle, Samiran Panda

**Affiliations:** ^1^HIV Drug Resistance Laboratory, Indian Council of Medical Research (ICMR)-National AIDS Research Institute, Pune, India; ^2^Division of Clinical Sciences, Indian Council of Medical Research (ICMR)-National AIDS Research Institute, Pune, India; ^3^Indian Council of Medical Research Headquarter, New Delhi, India

**Keywords:** HIV-1, Unnao, transmission cluster, evolution, phylodynamics

## Abstract

Certain rural and semiurban settings in the Unnao district, Uttar Pradesh, India observed an unprecedented increase in the detection of HIV cases during July 2017. Subsequent investigations through health camps and a follow-up case-control study attributed the outbreak to the unsafe injection exposures during treatment. In this study, we have undertaken a secondary analysis to understand the phylogenetic aspects of the outbreak-associated HIV-1 sequences along with the origin and phylodynamics of these sequences. The initial phylogenetic analysis indicated separate monophyletic grouping and there was no mixing of outbreak-associated sequences with sequences from other parts of India. Transmission network analysis using distance-based and non-distance-based methods revealed the existence of transmission clusters within the monophyletic Unnao clade. The median time to the most recent common ancestor (tMRCA) for sequences from Unnao using the pol gene region was observed to be 2011.87 [95% highest posterior density (HPD): 2010.09–2013.53], while the estimates using envelope (env) gene region sequences traced the tMRCA to 2010.33 (95% HPD: 2007.76–2012.99). Phylodynamics estimates demonstrated that the pace of this local epidemic has slowed down in recent times before the time of sampling, but was certainly on an upward track since its inception till 2014.

## Introduction

Over the last decade, the HIV epidemic in India has witnessed a decline, however, there are variations across the regions, states, and districts. Uttar Pradesh is one of the most populous states with the HIV prevalence currently below the national average (NACO, 2020).

In July 2017 the integrated counseling and testing center (ICTC) in Unnao District Hospital in Uttar Pradesh (UP), India, reported increased detection of HIV among its attendees. In response to these trends, health camps were organized from November 2017 to April 2018 in surrounding villages of Bangarmau block, Unnao, Uttar Pradesh to facilitate HIV testing. This was followed up by a case-control study to identify factors associated with HIV infection in the rural and semiurban study settings in the district of Unnao. During this investigation, 33 cases and 125 controls were enrolled. Cases were individuals, who detected HIV positive during November 2017–April 2018 from three locations namely Premganj, Karimuddinpur, and Chakmeerapur. Controls were enrolled from the same geographical setting and tested HIV negative either in health camps or at ICTC centers from where the cases were detected. Using clinical and laboratory investigations, this study could establish the possibility of iatrogenic transmission of HIV-1 due to unsterile injection equipment usage in a therapeutic setting. Among those detected HIV seroreactive, sequencing was carried out for 14 samples that had a detectable viral load. Phylogenetic analysis of these 14 sequences highlighted the monophyletic grouping (Patil et al., 2020).

Viral sequence data have been known to provide meaningful insights into infection transmission networks, origin, history, and dynamics of an epidemic (Pybus and Rambaut, 2009; Frost and Volz, 2010; Stadler et al., 2012, 2013; Ragonnet-Cronin et al., 2013; Campbell et al., 2021). Phylogenetic and phylodynamic analysis have been applied earlier in the case of localized HIV-1 outbreaks to unearth the transmission patterns, history, and demographic reconstruction of an outbreak (Rouet et al., 2018; Abidi et al., 2021). These fascets with respect to the HIV-1 outbreak from Unnao were largely unknown. In this study, we have performed detailed phylogenetic and phylodynamic analysis to investigate the transmission network patterns along with the evolutionary and demographic history of the aforementioned outbreak in the district of Unnao.

## Materials and Methods

### Ethical Consideration

This study was undertaken as a secondary analysis of an earlier study published by our group, which was approved by the Institutional Ethics Committee (Ethical approval reference: NARI/SP/17-18/3366 dated 29th May 2018) (Patil et al., 2020). HIV-1 sequences were available in the HIV sequence database (https://www.hiv.lanl.gov/) during the initiation of the current analysis (MW016009-MW016022 and MW016023-MW016034).

### Dataset

HIV-1 pol gene region (MW016009-MW016022) and envelope region (MW016023-MW016034) sequences representing the Unnao outbreak were retrieved from the Los Alamos HIV sequence database. Reference datasets representing rest of the India were also downloaded from Los Alamos HIV sequence database (https://www.hiv.lanl.gov/). For the Indian HIV-1 subtype C pol gene region reference dataset we downloaded HIV-1 pol gene region sequences spanning HXB2 coordinates 2253–3283, one sequence per patient. With these parameters, we could retrieve 1,440 sequences. After the removal of 30 problematic sequences, 1,410 sequences were retained (Supplementary Table 1). Using the same criteria we could retrieve 285 Indian HIV-1 subtype C env gene region sequences spanning HXB2 coordinates 6938-7366 (Supplementary Table 2).

### Phylogenetic Tree Construction

The phylogenetic relatedness of pol gene sequences from Unnao (MW016009-MW016022) with pan India HIV-1 C pol region sequences was assessed by constructing a maximum-likelihood phylogenetic tree. Reference sequences (*n* = 1,410) were aligned with 14 Unnao sequences using MAFFT v7.450 (Katoh et al., 2002). All major drug resistance mutation sites (Protease codons−30, 32, 33, 46, 47, 48, 50, 54, 76, 82, 84, 88, 90 and reverse transcriptase codons−41, 65, 67, 69, 70, 74, 100, 101, 103, 106, 115, 138, 151, 181, 184, 188, 190, 210, 215, 219, 230) were deleted before performing further analyses. This alignment of 1,424 sequences was used to construct a maximum-likelihood (ML) phylogenetic tree using a general time-reversible plus gamma (GTR + G) nucleotide substitution model and 1,000 bootstrap replicates with the help of the IQ-Tree v2.0 platform (Minh et al., 2020). The final tree was visualized and annotated with the help of Interactive Tree of Life (iTOL) v 5 (Letunic and Bork, 2021).

Details for the phylogenetic tree using corresponding env gene region sequences are provided in the Supplementary Material.

### Transmission Cluster Identification and Network Visualization

The initial pol gene region ML tree was employed to identify the transmission clusters. For distance-based approaches, we employed the genetic distance thresholds of 1.5 and 3.0%, mainly to identify the recently growing and long-lived transmission clusters (Novitsky et al., 2020).

Putative transmission clusters were identified with the help of the cluster picker v1.2.5 tool (Ragonnet-Cronin et al., 2013). Clusters were defined based on clades having ≥ 90% bootstrap node support and maximum pairwise intracluster genetic distance of <3.0% for grouping of 2 or more than 2 sequences. Recent transmission clusters were identified using a genetic distance threshold of 1.5% and ≥90% bootstrap node support for the grouping of ≥2 sequences (Kaye et al., 2008; Li et al., 2016; Chen et al., 2018; Dennis et al., 2020).

For the purpose of constructing the transmission network using Microbe Trace (Campbell et al., 2021), the initial ML tree was employed. The information regarding the Unnao sequences was provided as the node list. Transmission networks were constructed using the genetic distance thresholds of 3 and 1.5% where minimum cluster size was set as the grouping of ≥2 sequences.

We also employed the Phydelity (https://github.com/alvinxhan/Phydelity) to perform the cluster sensitivity analysis using pol gene region sequences (Han et al., 2019).

### The Bayesian Evolutionary Analysis

For the Bayesian analysis using pol gene, the dataset constituted the sequences grouping with Unnao sequence clade, extracted from initial pol gene region ML tree. We ensured that all of these sequences possessed the tip dates. Before commencing the Bayesian analysis, these sequences were subjected to the estimation of the molecular clock-like temporal signal using TempEst v1.5.3 software (Rambaut et al., 2016). After the removal of outlier sequences, the final dataset contained a total of 114 sequences including sequences from Unnao.

In the resulting dataset, a general time-reversible substitution model with a gamma-distributed rate of variation and a proportion of invariant sites (GTR + G + I) was observed to be a best-fitting model as assessed by jModelTest 2.1 (Darriba et al., 2012). The Bayesian maximum clade credibility (MCC) tree was constructed using BEAST v1.10.4 (Drummond and Rambaut, 2007) with the implementation of the BEAGLE library (Ayres et al., 2012). An uncorrelated relaxed lognormal parameter with GTR + G + I substitution model and the Bayesian skyline tree prior was employed for BEAST analysis. Normal distribution with a mean of 1.65 × 10^−3^ was used as the prior for the mean rate with the upper bound restricted to 1.9 × 10^−3^ while the lower bound was restricted to 1.4 × 10^−3^ (Patino-Galindo and Gonzalez-Candelas, 2017). BEAST analysis was carried out for 300 million states with sampling at every 30,000 states. The final BEAST parameters were assessed for convergence using Tracer v 1.7.1 with a 10% burn-in (Rambaut et al., 2018). The convergence for each parameter was defined as satisfactory when the effective sampling size (ESS) for a particular parameter was observed to be more than 200. Tree Annotator v 1.10.4 was employed to generate a final annotated tree, a 10% burn-in was used and the resultant MCC tree was visualized using Fig Tree v 1.4.4.

Details for the Bayesian evolutionary analysis using corresponding env sequences are provided in the Supplementary Material.

### Demographic Reconstruction

The Bayesian skyline plot (Drummond et al., 2005) was constructed for Unnao sequence clade (pol gene region, *n* = 14) using BEAST v2.6.3 (Bouckaert et al., 2019) with HKY + G substitution model, best fit model as assessed by jModelTest 2.1 and a strict molecular clock model since the dataset is homochromous, with a clock rate of 1.62 × 10^−3^. The mean tMRCA date of the Unnao cluster estimated in the earlier analysis was used as the prior to set the tree height using log-normal distribution (*M* = 1.82; *S* = 0.15) which places the median height close to the tMRCA estimates inferred for these sequences. Simulations were carried out for 100 million states with sampling at every 10,000 states. The final BEAST parameters were assessed for convergence using Tracer v 1.7.1 with a 10% burn-in.

Estimation of the reproductive number was also performed using pol gene region sequences (*n* = 14) with the help of BEAST v2.6.3. For this analysis, we employed the HKY + G substitution model, the best fit model as assessed by jModelTest 2.1, and a strict molecular clock model with a clock rate of 1.62 × 10^−3^. Birth-Death Skyline Contemporary (BDSKY) was used as the tree prior since these sequences were sampled at the same point in time. The following prior distributions of the BDSKY model were employed: log-normal (0; 1.25) for effective reproductive number R placing median at 1 with most weight below 7.82, log-normal (0; 1.25) for becoming noninfectious rate-setting quantile for the infectious period between 31 days and 11.5 years with 95% quantile below 7.82 years, beta (1.0; 10.0) for sampling probability placing most weight below 0.25 (between 0.0025 and 0.25) reflecting minority of the sampled cases. For origin prior we used the normal distribution (mean = 8.0; sigma = 1.0) setting for the origin of the epidemic before the tMRCA of the Unnao cluster. The mean tMRCA date of the Unnao cluster estimated in the earlier analysis was used as the prior to set the tree height using log-normal distribution (*M* = 1.82; *S* = 0.15). Simulations were carried out for 100 million states with sampling at every 10,000 states. The final BEAST parameters were assessed for convergence using Tracer v 1.7.1 with a 10% burn-in. The results of the BDSKY analysis were plotted in the R environment with the help of the bdskytools package.

## Results

### Demographic Details

The majority of the sequences analyzed in this study represented the cases from Premganj Township (10 out of 14). While out of the remaining 4, two were from Chakmeerapur and one from Kirvidyapur and Karimuddinpur each. The median age calculated for these cases was 41.5 years with the minimum age being 18 years while the maximum age recorded was 72 years. Out of these 14 cases, 9 were females, while 5 were males. All the 14 cases were married, which included 2 sets of couples (Couple-1−18-5451_010711 and 18-5479_010901, Couple-2−18-5478_010891, and 18-5482_011100).

### Phylogenetic Relatedness to Sequences From Other Parts of India

**Figure 1:**
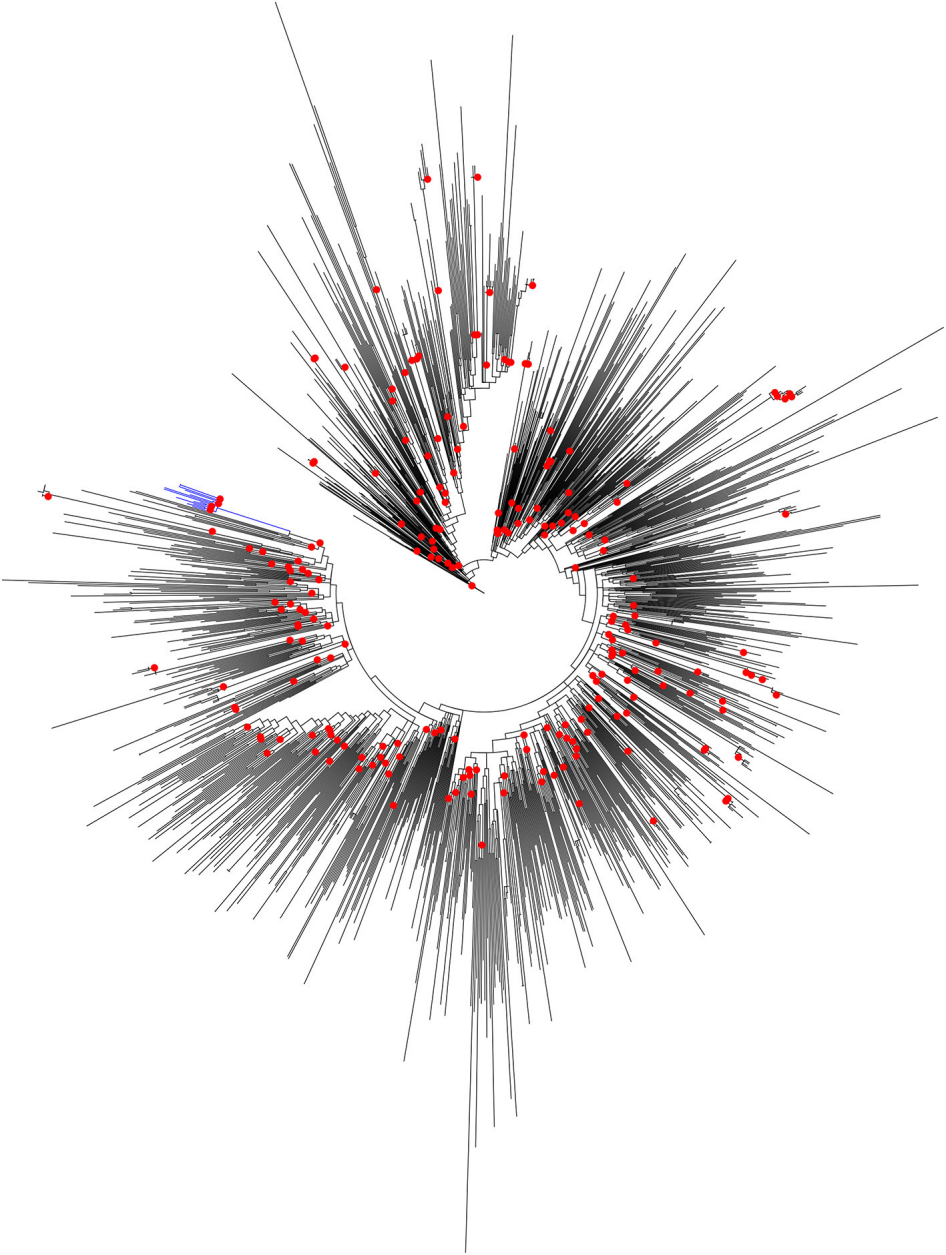
Maximum-likelihood phylogenetic tree depicting the phylogenetic relatedness of Unnao HIV-1 C pol gene sequences with other Indian HIV-1 C pol gene sequences available in Los Alamos HIV sequence database. Only bootstrap values of 100 are depicted with red circles at respective nodes. The node representing 14 Unnao sequences is depicted in blue color.

It was reported earlier that the HIV-1 pol region sequences from Unnao (*n* = 14) segregated with HIV-1 C sequences within the HIV-1 subtype reference sequence alignment (*n* = 40) (Patil et al., 2020). However, since these sequences were linked to the local HIV-1 outbreak we thought it would be worth analyzing them in the context of HIV-1 C sequences from other parts of the country. To address this, the maximum likelihood phylogenetic tree (Figure 1) was constructed. Sequences from Unnao did not mix with the other Indian sequences, and rather formed a single well-supported (Bootstrap = 98) monophyletic clade. This monophyletic clade consisted of some sequences with relatively longer branch lengths while a few of them had shorter branch lengths. Moreover, the ancestral branch separating the Unnao clade from the rest of the Indian sequences was observed to be relatively long with the particular ancestral node having bootstrap support of 100 (Supplementary Figure 1). The phylogenetic tree constructed for the envelope gene region for 11 out of 14 corresponding sequences highlighted the similar, distinctive monophyletic grouping of sequences from Unnao (Supplementary Figure 2).

### Transmission Cluster Analysis

Putative transmission clusters with respect to sequences from Unnao were identified using an initial ML tree constructed using 1,424 HIV-1 pol gene sequences. With the help of cluster picker v1.2.5 tool, using the analyses parameters of 90% bootstrap support for nodes with an intracluster genetic distance threshold of <3%, of the 1,424 sequences, 347 (24.36%) segregated into 91 transmission clusters with sizes ranging between 2 and 14 sequences. The intracluster genetic distances for these 91 clusters ranged from 0 to 2.91%.

**Figure 2:**
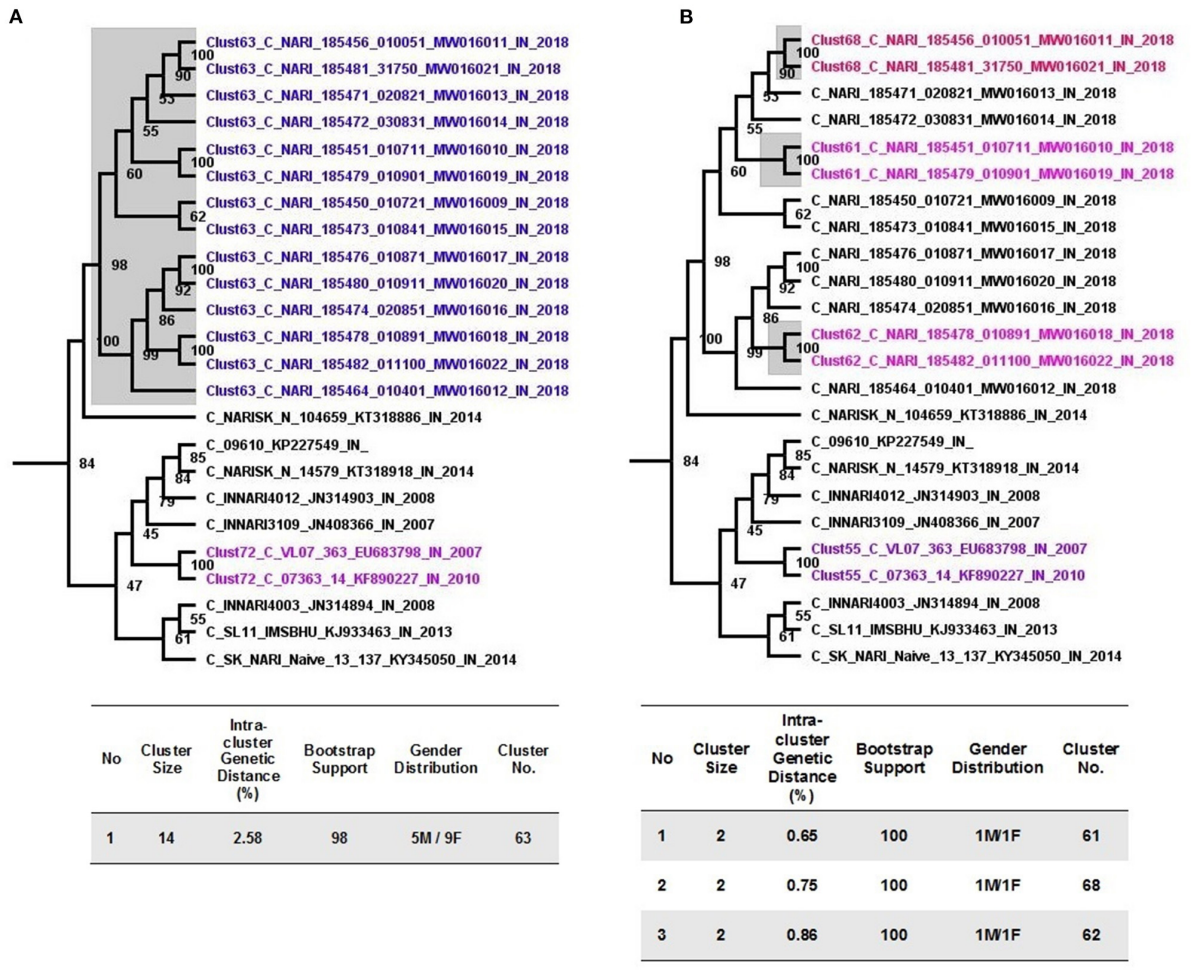
Cluster picker analysis. **(A)** Unnao transmission cluster node (highlighted gray) from an annotated tree obtained after cluster picker analysis at a genetic distance of 3%. Adjoining tables enlist the properties of the cluster. **(B)** Recent transmission events (gray highlighted) are depicted within the Unnao transmission cluster at a genetic distance threshold of 1.5%. The adjoining table enlists the properties of the individual cluster.

Pertaining to 14 sequences from Unnao, at a 3% genetic distance threshold, they clustered within a single transmission cluster (Figure 2A). Moreover, this cluster was observed to be the largest cluster out of 91 clusters identified. Unnao transmission cluster was observed to have strong bootstrap support of 98 and a mean intracluster genetic distance of 2.58%. This single transmission cluster constituted 5 males and 9 females.

When more stringent criteria were applied for the detection of recent transmission clusters as described in the methodology, the number of clusters for the complete dataset decreased to 72 as expected. In the case of sequences from Unnao (Figure 2B), out of 14, only 6 sequences were segregated into 3 clusters, each having 2 sequences with all the clusters having bootstrap support of 100. Within each of these clusters, 1 was male and 1 female. For these 3 clusters, the intracluster genetic distance was observed to be 0.65, 0.75, and 0.86%, respectively.

**Figure 3:**
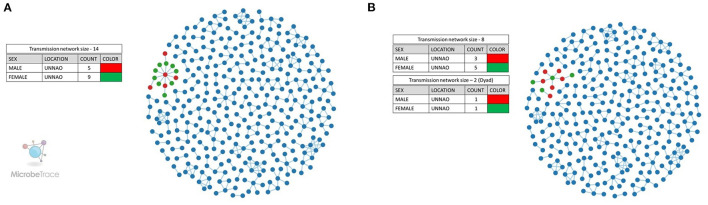
Transmission network visualization by Microbe Trace. **(A)** Transmission network at a genetic distance threshold of 3%. **(B)** Transmission network at a genetic distance threshold of 1.5%. Legends represent the colors used for the annotation of node sex for Unnao sequences where red represents the male while green represents the female. The blue-colored nodes represent the sequences from the rest of India.

We also employed the Microbe Trace tool to visualize putative transmission links. Microbe Trace visualization revealed that at a genetic distance threshold of 3%, sequences from Unnao belonged to a single transmission network (Figure 3A). As in the case of the cluster picker when more stringent criteria of 1.5% genetic distance were applied, the Unnao transmission network harboring all 14 sequences segregated into two networks of eight and two sequences (dyad), respectively (Figure 3B). Four sequences were observed to have no linking partners. The transmission network containing eight sequences had four sequences (C_NARI_185451_010711_MW016010_IN_2018, C_NARI_185479_010901_MW016019_IN_2018, C_NARI_185478_010891_MW016018_IN_2018, and C_NARI_185482_011100_MW016022_IN_2018) observed to be a part of the clusters identified by cluster picker analysis at a 1.5% genetic distance threshold. Two sequences (C_NARI_185456_010051_MW016011_IN_2018 and C_NARI_185481_31750_MW016021_IN_2018) were shown to be constituting a single separate cluster by cluster picker segregated as dyad (*n* = 2) transmission. There were 3 males and 5 females within the transmission network of eight sequences while the dyad transmission event had 1 male and 1 female. The remaining 4 sequences (C_NARI_185450_010721_MW016009_IN_2018, C_NARI_185471_020821_MW016013_IN_2018, C_NARI_185473_010841_MW016015_IN_2018, and C_NARI_185474_020851_MW016016_IN_2018) were not visible at this threshold indicating no linked partnerships for these sequences and therefore possibly represent the singleton transmissions.

Transmission cluster analysis using Phydelity (https://github.com/alvinxhan/Phydelity) showed that HIV-1 pol sequences from Unnao were segregated into two clusters. Out of these two clusters larger cluster included the majority of sequences (*n* = 10), while 4 were part of the smaller cluster (Supplementary Figure 3).

### Dating of the Unnao Transmission Cluster

To infer the age of the node for the putative Unnao transmission cluster, these sequences along with sequences grouping with this cluster were subjected to the Bayesian Markov Chain Monte Carlo evolutionary analysis using BEAST v1.10.4. The molecular clock-like pattern for these sequences was confirmed by performing regression analysis to estimate the correlation between sampling dates and root to tip genetic divergence using TempEst v1.5.3. After removal of the incongruent and outlier sequences, the dataset consisted of 114 sequences representing the years 2004–2018. Regression analysis showed that the correlation coefficient for this dataset was 0.76 with *r*^2^ of 0.57, suggesting a strong clock-like pattern with an estimated substitution rate of 1.37 × 10^−3^ per site per year.

**Figure 4:**
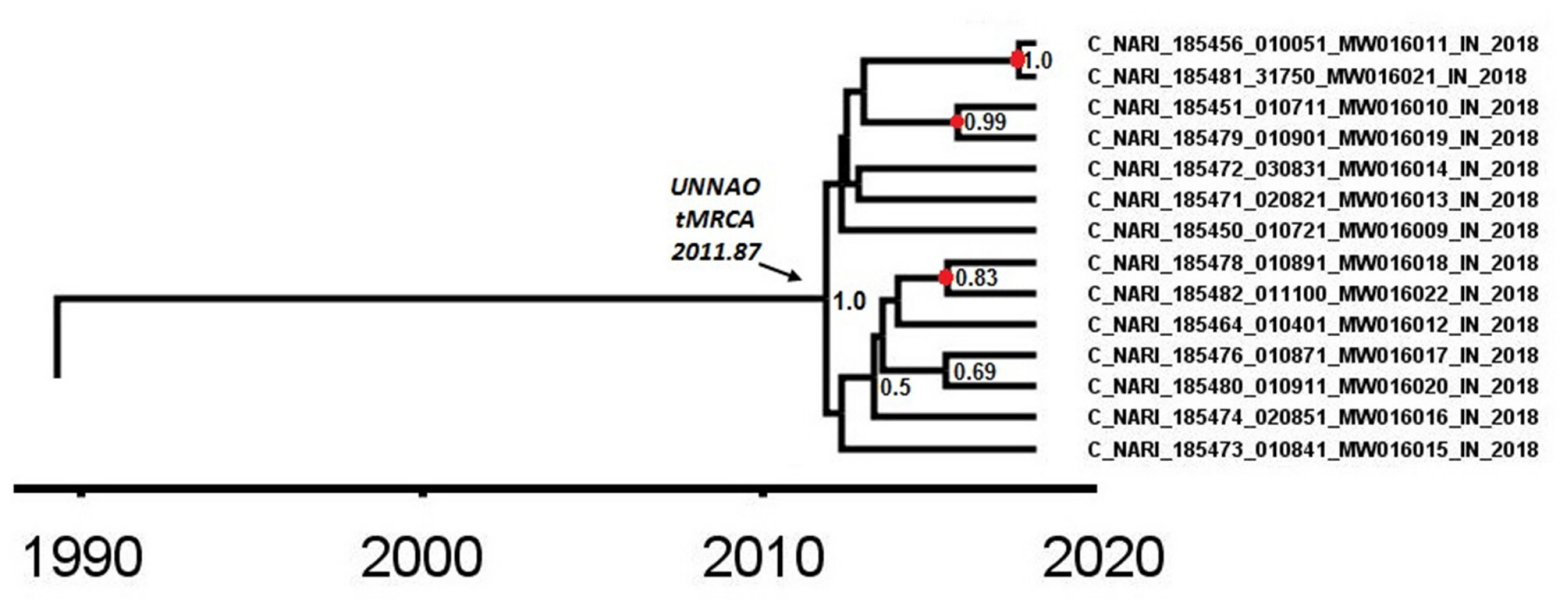
A node from a pol gene region MCC tree was obtained after BEAST analysis. The node depicted by the arrow highlights the time (2011.87) of the most recent common ancestor for the Unnao transmission cluster. The nodes represented by the red circles depict the meantime of the recent transmission events identified by the cluster picker. Posterior probability values of ≥0.5 are displayed.

As observed by Tracer analysis, most of the BEAST-derived parameters had an ESS of more than 200 indicating reliable convergence and sufficient sampling. With an estimated mean rate of 1.69 × 10^−3^ (95% HPD: 1.44 × 10^−3^-1.79 × 10^−3^) substitutions per site per year, the mean age of a node representing the Unnao transmission cluster (Figure 4) with a posterior probability of 1 was dated to 2011.87 (95% HPD: 2010.09–2013.53). The nodes identified as the three recent transmission events by the cluster picker within the Unnao cluster were dated to 2015.42 (95% HPD: 2014.15–2017.23), 2015.7 (95% HPD: 2014.01–2017.24), and 2017.52 (95% HPD: 2016.59–2018.0) with posterior probability supports of 0.83, 0.99, and 1, respectively.

A similar analysis was undertaken for env gene region sequences (*n* = 11) as described in the Supplementary Methods. The tMRCA estimates using env gene region were 2010.33 (95% HPD: 2007.76–2012.99) with an estimated mean rate of 4.46 × 10^−3^ (95% HPD: 3.89 × 10^−3^-5.03 × 10^−3^) substitutions per site per year (Supplementary Figure 4).

### Demographic History and Reproductive Number Estimates for the Outbreak

**Figure 5:**
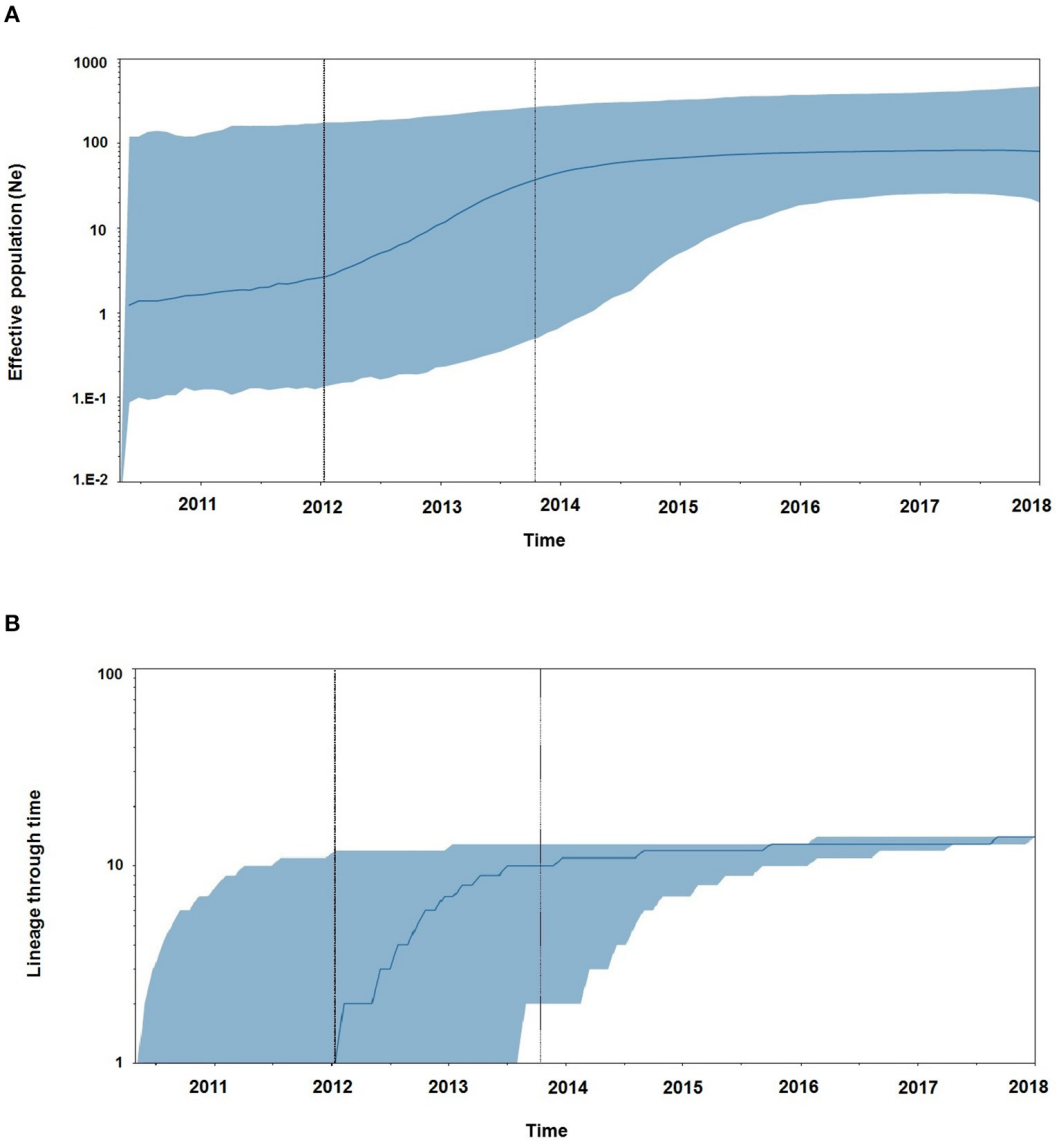
**(A)** The Bayesian skyline plots derived using pol gene region representing the estimates of effective population size in a log scale over time for the Unnao transmission cluster. The solid line represents the median estimates while the gray-shaded area indicates the 95% HPD. Vertical lines represent the estimated lower and median 95% HPD pertaining to time scale. **(B)** Lineage through time plot displaying the cumulative number of linages (infections) over time.

**Figure 6:**
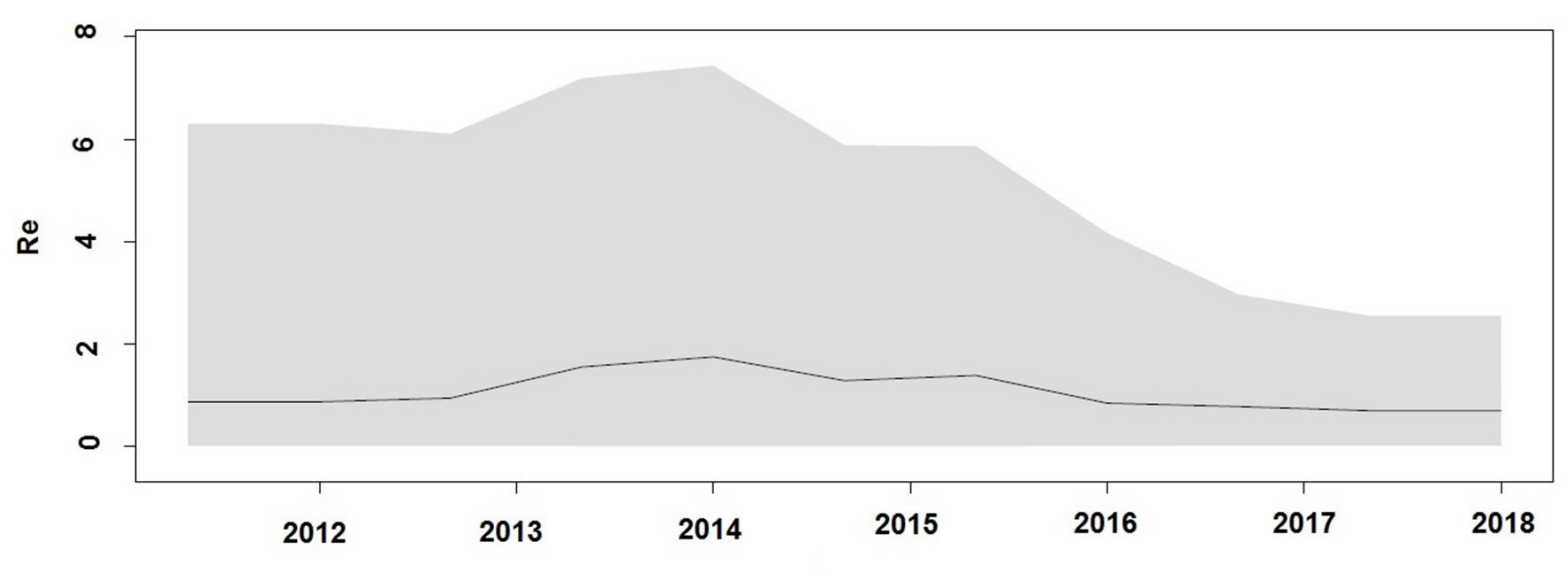
Birth death Skyline plot derived using pol gene region demonstrating the estimates of median effective reproductive number (*R*_*e*_) over time.

Demographic reconstruction of Unnao sequence clade using pol gene region sequences displayed steady exponential growth in effective population size (Figure 5A) and the number of infections (Figure 5B) from the beginning of the outbreak (2011) till the start of the year 2014. During this time frame, the effective population experienced a growth of over one log. The effective population size was observed to be stabilizing around early 2014, which was further reflected in the stationary phase between 2014 and 2018. The BDSKY analysis (Figure 6) showed that the median effective reproductive number (*R*_*e*_) gradually went on increasing till the start of the year 2014. Between 2012 and 2014 the *R*_*e*_ was consistently above the 1 and reached its peak by the start of 2014. Thereafter, until late 2014 though the *R*_*e*_ followed a downward trend it remained above 1.0. Thereafter it witnessed a slight rise followed by a steep fall which continued until the start of 2016. From 2016 onward till 2018 *R*_*e*_ witnessed a stationary phase.

## Discussion

The case-control study concluded that experiencing unsafe injecting practices in care settings was strikingly high among people living with HIV compared to those who did not contract HIV. It also underlined further that the role of such unsafe injecting could not be ruled out as a factor associated with HIV transmission in the study area. Phylogenetic tree of HIV-1 pol gene region sequences in the backdrop of minimal HIV-1 subtype reference sequences showed that the study sequences were monophyletic and clustered with Indian HIV-1 C reference sequences (Patil et al., 2020).

During this secondary analysis, the insights into the evolutionary and phylodynamic aspects of these outbreak-associated sequences were addressed. Transmission cluster structure identification along with detailed phylogenetic and phylodynamic analysis has been shown to assist in inferring the evolutionary context of an HIV-1 outbreak (Rouet et al., 2018; Abidi et al., 2021). In this analysis, we have employed the detailed phylogenetic, transmission network reconstruction and the Bayesian phylodynamic approach to infer the evolutionary and demographic parameters of this particular outbreak from Unnao, India.

In our analysis, ML phylogenetic tree reconstruction for pol and env gene regions demonstrated the monophyletic nature of Unnao sequences without any mixing with non-outbreak-associated sequences. While our ML tree analysis was carried out against the backdrop of available pan-India reference sequences for respective genomic regions, these observations suggested a close epidemiological linkage between the sequences from Unnao and thereby indicating the possible localized outbreak-associated clustering pattern. Branch lengths of the sequences within the monophyletic Unnao clade reflected the varied genetic diversity. Considering that these sequences were associated with an outbreak due to unsafe injection use, it is important to note that these observations may indicate the different time points of virus introductions. To be precise sequences with longer branch lengths indicate infections taking place relatively earlier than those possessing shorter branch lengths. In addition, the relatively longer well-supported ancestral branch separating the Unnao sequence clade from the rest of the Indian sequences points toward the possibility of still unsampled transmissions of this viral strain or no transmission of this virus for a few years.

Transmission cluster identification using distance-based approaches at a liberal genetic distance threshold of 3%, along with a non-distance-based approach (Phydelity) highlighted the presence of single or at the most 2 transmission clusters within the monophyletic Unnao sequence clade. The key observation of the Phydelity-based transmission cluster structure was that out of 2 clusters, the largest cluster (cluster-1) contained 9 out of 10 sequences representing Premganj Township. Differences in cluster structures using two different approaches could be attributed to the different algorithms used by the approaches employed. Sequences from the individual couples (Couple-1−18-5451_010711_ and 18-5479_010901, Couple-2−18-5478_010891 and 18-5482_011100) were observed to be linked using both the approaches. Moreover, the analysis using Phydelity showed that both of these couples were part of a single cluster (Cluster-1). Overall, the crux of these findings alluded to linked virus transmissions having shared ancestry.

The Bayesian evolutionary analysis could further resolve that the tMRCA for sequences from Unnao could be dated back to timelines between the middle of 2010 and late 2011 using different genomic regions. Estimates of the Bayesian evolutionary analysis indicate an ongoing HIV-1 transmission quite a few years before confirming the HIV-1 seroreactive status (November 2017–April 2018). Once we could infer upon the tMRCA for outbreak-associated sequences, the pertinent question was how did this outbreak shape up with respect to effective population and viral lineages? Demographic reconstruction demonstrated that the number of infections over time (lineage through time) followed an upward trend between 2011 and 2014 as observed by the lineage through time plot, moreover the effective population size was also seen to be increasing exponentially during this period. Estimates of the effective reproductive number also suggested the increasing number of infections occurring between 2012 and 2014, where *R*_*e*_ was seen to be at its peak at the start of 2014. These observations indicate the possibility of increased transmission of this particular viral strain between the timeframe spanning years 2012 and 2014, which was followed by plateauing effect.

In addition to corroborating the monophyletic cladding of HIV-1 sequences associated with the Unnao outbreak (Patil et al., 2020), the current investigation could further resolve the transmission cluster structure, origin, and phylodynamics of these sequences, which was certainly the enigma in hindsight. The major limitation of our investigation is the analyses of the limited number of outbreak-associated sequences and perhaps also the use of informed prior distributions for substitution rate during the Bayesian analysis. Increased sampling in conjunction with an in-depth phylogenetic and phylodynamic analysis may further elaborate on the true width and depth of this outbreak. However, this extended secondary analysis in the backdrop of an earlier case-control study could provide further insights by bridging these findings with socio-clinical observations. From the public health point of view, in conjunction with the findings of the parent study, the current investigation would strongly advocate for the sensitization of the local community and emphasis on effective regulatory practices in healthcare settings.

In summary, using phylogenetic and evolutionary analysis we could establish that the outbreak-associated sequences were part of the linked transmission cluster sharing a common ancestor dating back to several years before the sampling timeframe. Phylodynamic aspects of the outbreak clearly indicated that even though there were instances of recent transmissions, the pace of the epidemic had certainly slowed down in the recent past before sampling, i.e., November 2017–April 2018.

## Data Availability Statement

The original contributions presented in the study are included in the article/Supplementary Material, further inquiries can be directed to the corresponding author/s.

## Author Contributions

AP: conceptualization, methodology, analysis, interpretation, original draft writing, review, and editing. SK and SPan: review, editing, and supervision. AR, SPat, and SG: data collection, review, and editing. All authors contributed to the article and approved the submitted version.

## Conflict of Interest

The authors declare that the research was conducted in the absence of any commercial or financial relationships that could be construed as a potential conflict of interest.

## Publisher's Note

All claims expressed in this article are solely those of the authors and do not necessarily represent those of their affiliated organizations, or those of the publisher, the editors and the reviewers. Any product that may be evaluated in this article, or claim that may be made by its manufacturer, is not guaranteed or endorsed by the publisher.
