# Supplementary material for: Exploring the Evolutionary History and Phylodynamics of Human Immunodeficiency Virus Type 1 Outbreak From Unnao, India Using Phylogenetic Approach
Source: Front Microbiol. 2022 May 18;13:848250. doi: 10.3389/fmicb.2022.848250 (PMC9158528; doi:10.3389/fmicb.2022.848250)
Supplement: Supplementary file 1 [file Data_Sheet_1.docx]

**Supplementary Methods**

**Phylogenetic tree construction for envelop gene region**

Sequences from Unnao (n=11) were combined with the reference sequences (n=285). MAFFT v7.450 was used to align the sequences. Phylogenetic tree was constructed using general time- reversible plus gamma (GTR+G) nucleotide substitution model and 1000 bootstrap replicates with the help of IQ-Tree v2.0 platform. The final tree was visualized and annotated with the help of Interactive Tree of Life (iTOL) v 5.

**Transmission cluster identification using Phydelity**

Sensitivity analysis for transmission clusters identified using distance based approaches (cluster picker v1.2.5 and Microbe Trace) was performed with help of Phydelity (<https://github.com/alvinxhan/Phydelity> ). Phydelity identifies the clusters that are more closely related to each other without the application of any distance thresholds.

**Bayesian evolutionary analysis using envelop gene region sequences**

Reference sequence dataset for Bayesian phylogenetic tree using envelope gene region sequences, only sequences possessing tip dates were extracted from the reference sequences employed for the initial ML tree. These reference sequences were combined with the sequences from Unnao. Molecular clockliness for the envelope gene region dataset was assessed with the help of TempEst v1.5.3. After removal of the outlier sequences the final dataset consisted 172 sequences including sequences from Unnao. The final dataset consisted 161 reference sequences and 11 sequences from Unnao. MCC tree was constructed using uncorrelated relaxed lognormal parameter with GTR+G+I substitution model and Bayesian skyline tree prior. Normal distribution with mean of 4.0 x 10^-3^ was used as the prior for mean rate with upper bound restricted to 5.0x 10-3 while the lower bound was restricted to 3.0 x 10^-3^.

BEAST analysis was carried out for 300 million states with sampling at every 30000 states. The final BEAST parameters were assessed for convergence using Tracer v 1.7.1 with a 10% burn-in. The convergence for each parameter was defined as satisfactory when the effective sampling size (ESS) for particular parameter was observed to be more than 200. Tree Annotator v 1.10.4 was employed to generate a final annotated tree, a 10% burn-in was used and the resultant MCC tree was visualized using Fig Tree v 1.4.4

**Supplementary Figures**

**Supplementary Figure 1.** Sub-tree from maximum likelihood phylogenetic tree displaying the branch lengths for sequences from Unnao (Blue) and branch length for the ancestral branch separating Unnao sequences from rest of the Indian sequences.

**Supplementary Figure 2.** Maximum-likelihood phylogenetic tree depicting the phylogenetic relatedness of Unnao HIV-1 C env gene sequences with other Indian HIV-1 C env gene sequences available in Los Alamos HIV sequence database. Only bootstrap values of 100 are depicted with red circles at respective nodes. Node representing 11 Unnao sequences is depicted in blue colour.

**Supplementary Figure 3.** Transmission cluster analysis performed using Phydelity (<https://github.com/alvinxhan/Phydelity> ). The node representing Unnao monophyletic clade is displayed. Transmission cluster structure shows two clusters, cluster-1 (Cyan) encompassing 10 sequences while cluster-2 (Purple) with 4 sequences.

**Supplementary Figure 4.** A node from env gene region MCC tree obtained after BEAST analysis. The node depicted by the arrow highlights the time (2010.33) of the most recent common ancestor for Unnao transmission cluster. Posterior probability values of ≥ 0.5 are displayed.

Supplementary Table 1 – Reference sequences used for pol gene region ML tree

| No | Accession | Subtype | Country | Sampling Year | Sampling City |
| --- | --- | --- | --- | --- | --- |
| 1 | AB023804 | C | INDIA | 1993 | Not Available |
| 2 | AF067154 | C | INDIA | 1993 | Pune |
| 3 | AF067157 | C | INDIA | 1993 | Pune |
| 4 | AF067158 | C | INDIA | 1993 | Pune |
| 5 | AF067159 | C | INDIA | 1994 | Pune |
| 6 | AF286223 | C | INDIA | 1994 | Pune |
| 7 | AY713414 | C | INDIA | 1994 | Not Available |
| 8 | AF067155 | C | INDIA | 1995 | Pune |
| 9 | AF286231 | C | INDIA | 1998 | T'Nagar Madras |
| 10 | AF286232 | C | INDIA | 1998 | Churachandpur |
| 11 | AY049708 | C | INDIA | 1999 | GUJARAT |
| 12 | AY746362 | C | INDIA | 1999 | Pune |
| 13 | AY746365 | C | INDIA | 1999 | Pune |
| 14 | AY746368 | C | INDIA | 1999 | Pune |
| 15 | AY746371 | C | INDIA | 1999 | Pune |
| 16 | AY746374 | C | INDIA | 1999 | Pune |
| 17 | AY746376 | C | INDIA | 1999 | Pune |
| 18 | AY746379 | C | INDIA | 1999 | Pune |
| 19 | AY746382 | C | INDIA | 1999 | Pune |
| 20 | AY746385 | C | INDIA | 1999 | Pune |
| 21 | AY746386 | C | INDIA | 1999 | Pune |
| 22 | AY739911 | C | INDIA | 2000 | Pune |
| 23 | AY739913 | C | INDIA | 2000 | Pune |
| 24 | KP109481 | C | INDIA | 2000 | Not Available |
| 25 | KP109482 | C | INDIA | 2000 | Not Available |
| 26 | KP109485 | C | INDIA | 2000 | Not Available |
| 27 | EF469243 | C | INDIA | 2003 | Bangalore |
| 28 | DQ826595 | C | INDIA | 2004 | Pune |
| 29 | DQ826596 | C | INDIA | 2004 | Pune |
| 30 | DQ826597 | C | INDIA | 2004 | Pune |
| 31 | DQ826598 | C | INDIA | 2004 | Pune |
| 32 | DQ826599 | C | INDIA | 2004 | Pune |
| 33 | DQ826600 | C | INDIA | 2004 | Pune |
| 34 | DQ826601 | C | INDIA | 2004 | Pune |
| 35 | DQ826602 | C | INDIA | 2004 | Pune |
| 36 | DQ826603 | C | INDIA | 2004 | Pune |
| 37 | DQ826604 | C | INDIA | 2004 | Pune |
| 38 | DQ826605 | C | INDIA | 2004 | Pune |
| 39 | DQ826606 | C | INDIA | 2004 | Pune |
| 40 | DQ826607 | C | INDIA | 2004 | Pune |
| 41 | DQ826608 | C | INDIA | 2004 | Pune |
| 42 | DQ826609 | C | INDIA | 2004 | Pune |
| 43 | DQ826610 | C | INDIA | 2004 | Pune |
| 44 | DQ826611 | C | INDIA | 2004 | Pune |
| 45 | DQ826612 | C | INDIA | 2004 | Pune |
| 46 | DQ826613 | C | INDIA | 2004 | Pune |
| 47 | DQ826614 | C | INDIA | 2004 | Pune |
| 48 | DQ826615 | C | INDIA | 2004 | Pune |
| 49 | DQ826616 | C | INDIA | 2004 | Pune |
| 50 | DQ826617 | C | INDIA | 2004 | Pune |
| 51 | DQ826618 | C | INDIA | 2004 | Pune |
| 52 | DQ826619 | C | INDIA | 2004 | Pune |
| 53 | DQ826622 | C | INDIA | 2004 | Pune |
| 54 | DQ826623 | C | INDIA | 2004 | Pune |
| 55 | DQ826625 | C | INDIA | 2004 | Pune |
| 56 | DQ826626 | C | INDIA | 2004 | Pune |
| 57 | DQ826628 | C | INDIA | 2004 | Pune |
| 58 | DQ826631 | C | INDIA | 2004 | Pune |
| 59 | DQ826632 | C | INDIA | 2004 | Pune |
| 60 | DQ826635 | C | INDIA | 2004 | Pune |
| 61 | DQ826636 | C | INDIA | 2004 | Pune |
| 62 | DQ826637 | C | INDIA | 2004 | Pune |
| 63 | DQ826638 | C | INDIA | 2004 | Pune |
| 64 | DQ826639 | C | INDIA | 2004 | Pune |
| 65 | DQ826640 | C | INDIA | 2004 | Pune |
| 66 | DQ826643 | C | INDIA | 2004 | Pune |
| 67 | DQ826644 | C | INDIA | 2004 | Pune |
| 68 | DQ826645 | C | INDIA | 2004 | Pune |
| 69 | DQ826646 | C | INDIA | 2004 | Pune |
| 70 | DQ826647 | C | INDIA | 2004 | Pune |
| 71 | DQ826648 | C | INDIA | 2004 | Pune |
| 72 | DQ826649 | C | INDIA | 2004 | Pune |
| 73 | DQ826650 | C | INDIA | 2004 | Pune |
| 74 | DQ826651 | C | INDIA | 2004 | Pune |
| 75 | DQ826652 | C | INDIA | 2004 | Pune |
| 76 | DQ826653 | C | INDIA | 2004 | Pune |
| 77 | DQ826654 | C | INDIA | 2004 | Pune |
| 78 | DQ826655 | C | INDIA | 2004 | Pune |
| 79 | DQ826656 | C | INDIA | 2004 | Pune |
| 80 | DQ826657 | C | INDIA | 2004 | Pune |
| 81 | DQ826658 | C | INDIA | 2004 | Pune |
| 82 | DQ826659 | C | INDIA | 2004 | Pune |
| 83 | DQ826660 | C | INDIA | 2004 | Pune |
| 84 | DQ826662 | C | INDIA | 2004 | Pune |
| 85 | DQ826663 | C | INDIA | 2004 | Pune |
| 86 | DQ826664 | C | INDIA | 2004 | Pune |
| 87 | DQ826665 | C | INDIA | 2004 | Pune |
| 88 | DQ826666 | C | INDIA | 2004 | Pune |
| 89 | DQ826667 | C | INDIA | 2004 | Pune |
| 90 | DQ826668 | C | INDIA | 2004 | Pune |
| 91 | DQ826669 | C | INDIA | 2004 | Pune |
| 92 | EF186926 | C | INDIA | 2004 | Pune |
| 93 | EF186928 | C | INDIA | 2004 | Pune |
| 94 | EF186929 | C | INDIA | 2004 | Pune |
| 95 | EF186930 | C | INDIA | 2004 | Pune |
| 96 | EF186931 | C | INDIA | 2004 | Pune |
| 97 | EF186932 | C | INDIA | 2004 | Pune |
| 98 | EF186933 | C | INDIA | 2004 | Pune |
| 99 | EF186935 | C | INDIA | 2004 | Pune |
| 100 | EF186936 | C | INDIA | 2004 | Pune |
| 101 | EF186938 | C | INDIA | 2004 | Pune |
| 102 | EF186940 | C | INDIA | 2004 | Pune |
| 103 | EF186942 | C | INDIA | 2004 | Pune |
| 104 | EF186954 | C | INDIA | 2004 | Pune |
| 105 | EF186960 | C | INDIA | 2004 | Pune |
| 106 | EF186962 | C | INDIA | 2004 | Pune |
| 107 | EF186965 | C | INDIA | 2004 | Pune |
| 108 | EF186966 | C | INDIA | 2004 | Pune |
| 109 | EF186967 | C | INDIA | 2004 | Pune |
| 110 | EF186969 | C | INDIA | 2004 | Pune |
| 111 | EF186974 | C | INDIA | 2004 | Pune |
| 112 | EF186976 | C | INDIA | 2004 | Pune |
| 113 | EF186982 | C | INDIA | 2004 | Pune |
| 114 | EF186986 | C | INDIA | 2004 | Pune |
| 115 | EF186987 | C | INDIA | 2004 | Pune |
| 116 | KF766537 | C | INDIA | 2004 | Tamilnadu |
| 117 | EU030410 | C | INDIA | 2005 | Not Available |
| 118 | EU030414 | C | INDIA | 2005 | Not Available |
| 119 | EU030416 | C | INDIA | 2005 | Not Available |
| 120 | EU030418 | C | INDIA | 2005 | Not Available |
| 121 | EU106124 | C | INDIA | 2005 | Not Available |
| 122 | EU106126 | C | INDIA | 2005 | Not Available |
| 123 | EU110053 | C | INDIA | 2005 | Not Available |
| 124 | EU110054 | C | INDIA | 2005 | Not Available |
| 125 | EU110055 | C | INDIA | 2005 | Not Available |
| 126 | EU110056 | C | INDIA | 2005 | Not Available |
| 127 | EU110057 | C | INDIA | 2005 | Not Available |
| 128 | EU110058 | C | INDIA | 2005 | Not Available |
| 129 | EU110059 | C | INDIA | 2005 | Not Available |
| 130 | EU110060 | C | INDIA | 2005 | Not Available |
| 131 | EU110061 | C | INDIA | 2005 | Not Available |
| 132 | EU110062 | C | INDIA | 2005 | Not Available |
| 133 | EU110063 | C | INDIA | 2005 | Not Available |
| 134 | EU110064 | C | INDIA | 2005 | Not Available |
| 135 | EU110065 | C | INDIA | 2005 | Not Available |
| 136 | EU110066 | C | INDIA | 2005 | Not Available |
| 137 | EU110067 | C | INDIA | 2005 | Not Available |
| 138 | EU110068 | C | INDIA | 2005 | Not Available |
| 139 | EU158865 | C | INDIA | 2005 | Pune |
| 140 | EU158866 | C | INDIA | 2005 | Pune |
| 141 | EU158867 | C | INDIA | 2005 | Pune |
| 142 | EU158868 | C | INDIA | 2005 | Pune |
| 143 | EU158869 | C | INDIA | 2005 | Pune |
| 144 | EU158870 | C | INDIA | 2005 | Pune |
| 145 | EU158871 | C | INDIA | 2005 | Pune |
| 146 | EU158872 | C | INDIA | 2005 | Pune |
| 147 | EU158873 | C | INDIA | 2005 | Pune |
| 148 | EU158874 | C | INDIA | 2005 | Pune |
| 149 | EU158875 | C | INDIA | 2005 | Pune |
| 150 | EU158876 | C | INDIA | 2005 | Pune |
| 151 | EU158877 | C | INDIA | 2005 | Pune |
| 152 | EU158878 | C | INDIA | 2005 | Pune |
| 153 | EU158879 | C | INDIA | 2005 | Pune |
| 154 | EU158880 | C | INDIA | 2005 | Pune |
| 155 | EU158881 | C | INDIA | 2005 | Pune |
| 156 | EU158882 | C | INDIA | 2005 | Pune |
| 157 | EU158885 | C | INDIA | 2005 | Pune |
| 158 | EU158886 | C | INDIA | 2005 | Pune |
| 159 | EU158887 | C | INDIA | 2005 | Pune |
| 160 | EU158888 | C | INDIA | 2005 | Pune |
| 161 | EU158889 | C | INDIA | 2005 | Pune |
| 162 | EU158890 | C | INDIA | 2005 | Pune |
| 163 | EU158891 | C | INDIA | 2005 | Pune |
| 164 | EU158892 | C | INDIA | 2005 | Pune |
| 165 | EU447776 | C | INDIA | 2005 | Pune |
| 166 | EU447778 | C | INDIA | 2005 | Pune |
| 167 | EU447779 | C | INDIA | 2005 | Pune |
| 168 | EU447780 | C | INDIA | 2005 | Pune |
| 169 | EU447781 | C | INDIA | 2005 | Pune |
| 170 | EU484322 | C | INDIA | 2005 | Pune |
| 171 | EU484323 | C | INDIA | 2005 | Pune |
| 172 | EU484324 | C | INDIA | 2005 | Pune |
| 173 | EU683780 | C | INDIA | 2005 | Not Available |
| 174 | JN634315 | C | INDIA | 2005 | Not Available |
| 175 | KF766540 | C | INDIA | 2005 | Tamilnadu |
| 176 | KF766541 | C | INDIA | 2005 | Tamilnadu |
| 177 | KU562078 | C | INDIA | 2005 | Not Available |
| 178 | EU030411 | C | INDIA | 2006 | Not Available |
| 179 | EU030412 | C | INDIA | 2006 | Not Available |
| 180 | EU030413 | C | INDIA | 2006 | Not Available |
| 181 | EU030415 | C | INDIA | 2006 | Not Available |
| 182 | EU037773 | C | INDIA | 2006 | Not Available |
| 183 | EU037774 | C | INDIA | 2006 | Not Available |
| 184 | EU106117 | C | INDIA | 2006 | Not Available |
| 185 | EU106118 | C | INDIA | 2006 | Not Available |
| 186 | EU106119 | C | INDIA | 2006 | Not Available |
| 187 | EU106120 | C | INDIA | 2006 | Not Available |
| 188 | EU110069 | C | INDIA | 2006 | Not Available |
| 189 | EU110070 | C | INDIA | 2006 | Not Available |
| 190 | EU110072 | C | INDIA | 2006 | Not Available |
| 191 | EU110073 | C | INDIA | 2006 | Not Available |
| 192 | EU110074 | C | INDIA | 2006 | Not Available |
| 193 | EU110075 | C | INDIA | 2006 | Not Available |
| 194 | EU110078 | C | INDIA | 2006 | Not Available |
| 195 | EU110079 | C | INDIA | 2006 | Not Available |
| 196 | EU110080 | C | INDIA | 2006 | Not Available |
| 197 | EU110083 | C | INDIA | 2006 | Not Available |
| 198 | EU683765 | C | INDIA | 2006 | Not Available |
| 199 | EU683766 | C | INDIA | 2006 | Not Available |
| 200 | EU683767 | C | INDIA | 2006 | Not Available |
| 201 | EU683768 | C | INDIA | 2006 | Not Available |
| 202 | EU683769 | C | INDIA | 2006 | Not Available |
| 203 | EU683772 | C | INDIA | 2006 | Not Available |
| 204 | EU683773 | C | INDIA | 2006 | Not Available |
| 205 | EU683776 | C | INDIA | 2006 | Not Available |
| 206 | EU683781 | C | INDIA | 2006 | Not Available |
| 207 | EU683782 | C | INDIA | 2006 | Not Available |
| 208 | EU683783 | C | INDIA | 2006 | Not Available |
| 209 | EU683784 | C | INDIA | 2006 | Not Available |
| 210 | EU683785 | C | INDIA | 2006 | Not Available |
| 211 | EU683789 | C | INDIA | 2006 | Not Available |
| 212 | EU683800 | C | INDIA | 2006 | Not Available |
| 213 | EU744912 | C | INDIA | 2006 | Not Available |
| 214 | EU744913 | C | INDIA | 2006 | Not Available |
| 215 | EU744914 | C | INDIA | 2006 | Not Available |
| 216 | HQ453364 | C | INDIA | 2006 | DELHI |
| 217 | HQ453367 | C | INDIA | 2006 | DELHI |
| 218 | HQ453368 | C | INDIA | 2006 | DELHI |
| 219 | HQ453384 | C | INDIA | 2006 | DELHI |
| 220 | HQ453389 | C | INDIA | 2006 | DELHI |
| 221 | HQ453390 | C | INDIA | 2006 | DELHI |
| 222 | EU037776 | C | INDIA | 2007 | Not Available |
| 223 | EU037778 | C | INDIA | 2007 | Not Available |
| 224 | EU037779 | C | INDIA | 2007 | Not Available |
| 225 | EU106121 | C | INDIA | 2007 | Not Available |
| 226 | EU106122 | C | INDIA | 2007 | Not Available |
| 227 | EU106123 | C | INDIA | 2007 | Not Available |
| 228 | EU106125 | C | INDIA | 2007 | Not Available |
| 229 | EU683752 | C | INDIA | 2007 | Not Available |
| 230 | EU683753 | C | INDIA | 2007 | Not Available |
| 231 | EU683754 | C | INDIA | 2007 | Not Available |
| 232 | EU683755 | C | INDIA | 2007 | Not Available |
| 233 | EU683756 | C | INDIA | 2007 | Not Available |
| 234 | EU683757 | C | INDIA | 2007 | Not Available |
| 235 | EU683758 | C | INDIA | 2007 | Not Available |
| 236 | EU683759 | C | INDIA | 2007 | Not Available |
| 237 | EU683761 | C | INDIA | 2007 | Not Available |
| 238 | EU683762 | C | INDIA | 2007 | Not Available |
| 239 | EU683763 | C | INDIA | 2007 | Not Available |
| 240 | EU683764 | C | INDIA | 2007 | Not Available |
| 241 | EU683770 | C | INDIA | 2007 | Not Available |
| 242 | EU683771 | C | INDIA | 2007 | Not Available |
| 243 | EU683775 | C | INDIA | 2007 | Not Available |
| 244 | EU683777 | C | INDIA | 2007 | Not Available |
| 245 | EU683778 | C | INDIA | 2007 | Not Available |
| 246 | EU683786 | C | INDIA | 2007 | Not Available |
| 247 | EU683787 | C | INDIA | 2007 | Not Available |
| 248 | EU683788 | C | INDIA | 2007 | Not Available |
| 249 | EU683790 | C | INDIA | 2007 | Not Available |
| 250 | EU683791 | C | INDIA | 2007 | Not Available |
| 251 | EU683792 | C | INDIA | 2007 | Not Available |
| 252 | EU683793 | C | INDIA | 2007 | Not Available |
| 253 | EU683795 | C | INDIA | 2007 | Not Available |
| 254 | EU683796 | C | INDIA | 2007 | Not Available |
| 255 | EU683797 | C | INDIA | 2007 | Not Available |
| 256 | EU683798 | C | INDIA | 2007 | Not Available |
| 257 | EU683799 | C | INDIA | 2007 | Not Available |
| 258 | EU683801 | C | INDIA | 2007 | Not Available |
| 259 | EU781834 | C | INDIA | 2007 | Not Available |
| 260 | EU781835 | C | INDIA | 2007 | Not Available |
| 261 | EU781836 | C | INDIA | 2007 | Not Available |
| 262 | EU781837 | C | INDIA | 2007 | Not Available |
| 263 | EU781838 | C | INDIA | 2007 | Not Available |
| 264 | EU781839 | C | INDIA | 2007 | Not Available |
| 265 | EU781840 | C | INDIA | 2007 | Not Available |
| 266 | FJ878937 | C | INDIA | 2007 | Mumbai |
| 267 | FJ878938 | C | INDIA | 2007 | Mumbai |
| 268 | FJ878939 | C | INDIA | 2007 | Mumbai |
| 269 | FJ878940 | C | INDIA | 2007 | Mumbai |
| 270 | FJ878941 | C | INDIA | 2007 | Mumbai |
| 271 | FJ878942 | C | INDIA | 2007 | Mumbai |
| 272 | FJ878943 | C | INDIA | 2007 | Mumbai |
| 273 | FJ878944 | C | INDIA | 2007 | Mumbai |
| 274 | FJ878945 | C | INDIA | 2007 | Mumbai |
| 275 | FJ878946 | C | INDIA | 2007 | Mumbai |
| 276 | FJ878947 | C | INDIA | 2007 | Mumbai |
| 277 | FJ878948 | C | INDIA | 2007 | Mumbai |
| 278 | FJ878949 | C | INDIA | 2007 | Mumbai |
| 279 | FJ878950 | C | INDIA | 2007 | Mumbai |
| 280 | FJ878951 | C | INDIA | 2007 | Mumbai |
| 281 | FJ878952 | C | INDIA | 2007 | Mumbai |
| 282 | FJ878953 | C | INDIA | 2007 | Mumbai |
| 283 | FJ878954 | C | INDIA | 2007 | Mumbai |
| 284 | FJ878955 | C | INDIA | 2007 | Mumbai |
| 285 | FJ878956 | C | INDIA | 2007 | Mumbai |
| 286 | FJ878957 | C | INDIA | 2007 | Mumbai |
| 287 | FJ878958 | C | INDIA | 2007 | Mumbai |
| 288 | FJ878959 | C | INDIA | 2007 | Mumbai |
| 289 | FJ878960 | C | INDIA | 2007 | Mumbai |
| 290 | FJ878961 | C | INDIA | 2007 | Mumbai |
| 291 | FJ878962 | C | INDIA | 2007 | Mumbai |
| 292 | FJ878963 | C | INDIA | 2007 | Mumbai |
| 293 | FJ878964 | C | INDIA | 2007 | Mumbai |
| 294 | FJ878965 | C | INDIA | 2007 | Mumbai |
| 295 | FJ878966 | C | INDIA | 2007 | Mumbai |
| 296 | FJ878967 | C | INDIA | 2007 | Mumbai |
| 297 | FJ878968 | C | INDIA | 2007 | Mumbai |
| 298 | FJ878969 | C | INDIA | 2007 | Mumbai |
| 299 | FJ878970 | C | INDIA | 2007 | Mumbai |
| 300 | FJ907462 | C | INDIA | 2007 | Not Available |
| 301 | FJ907463 | C | INDIA | 2007 | Not Available |
| 302 | FJ907464 | C | INDIA | 2007 | Not Available |
| 303 | FJ907466 | C | INDIA | 2007 | Not Available |
| 304 | FJ907467 | C | INDIA | 2007 | Not Available |
| 305 | FJ907469 | C | INDIA | 2007 | Not Available |
| 306 | FJ907470 | C | INDIA | 2007 | Not Available |
| 307 | FJ907471 | C | INDIA | 2007 | Not Available |
| 308 | FJ907472 | C | INDIA | 2007 | Not Available |
| 309 | FJ907473 | C | INDIA | 2007 | Not Available |
| 310 | FJ907478 | C | INDIA | 2007 | Not Available |
| 311 | FJ907479 | C | INDIA | 2007 | Not Available |
| 312 | FJ907480 | C | INDIA | 2007 | Not Available |
| 313 | FJ907485 | C | INDIA | 2007 | Not Available |
| 314 | FJ907488 | C | INDIA | 2007 | Not Available |
| 315 | FJ907497 | C | INDIA | 2007 | Not Available |
| 316 | GQ891861 | C | INDIA | 2007 | Not Available |
| 317 | HQ166841 | C | INDIA | 2007 | KAKINADA |
| 318 | HQ166842 | C | INDIA | 2007 | KAKINADA |
| 319 | HQ166843 | C | INDIA | 2007 | KAKINADA |
| 320 | HQ166844 | C | INDIA | 2007 | KAKINADA |
| 321 | HQ166845 | C | INDIA | 2007 | KAKINADA |
| 322 | HQ453365 | C | INDIA | 2007 | DELHI |
| 323 | HQ453366 | C | INDIA | 2007 | DELHI |
| 324 | HQ453370 | C | INDIA | 2007 | DELHI |
| 325 | HQ453371 | C | INDIA | 2007 | DELHI |
| 326 | HQ453372 | C | INDIA | 2007 | DELHI |
| 327 | HQ453373 | C | INDIA | 2007 | DELHI |
| 328 | HQ453374 | C | INDIA | 2007 | DELHI |
| 329 | HQ453375 | C | INDIA | 2007 | DELHI |
| 330 | HQ453376 | C | INDIA | 2007 | DELHI |
| 331 | HQ453379 | C | INDIA | 2007 | DELHI |
| 332 | HQ453380 | C | INDIA | 2007 | DELHI |
| 333 | HQ453381 | C | INDIA | 2007 | DELHI |
| 334 | HQ453386 | C | INDIA | 2007 | DELHI |
| 335 | HQ453387 | C | INDIA | 2007 | DELHI |
| 336 | HQ453388 | C | INDIA | 2007 | DELHI |
| 337 | JN408359 | C | INDIA | 2007 | Mumbai |
| 338 | JN408360 | C | INDIA | 2007 | Mumbai |
| 339 | JN408361 | C | INDIA | 2007 | Mumbai |
| 340 | JN408362 | C | INDIA | 2007 | Mumbai |
| 341 | JN408363 | C | INDIA | 2007 | Mumbai |
| 342 | JN408364 | C | INDIA | 2007 | Mumbai |
| 343 | JN408365 | C | INDIA | 2007 | Mumbai |
| 344 | JN408366 | C | INDIA | 2007 | Mumbai |
| 345 | JN408367 | C | INDIA | 2007 | Mumbai |
| 346 | JN408368 | C | INDIA | 2007 | Mumbai |
| 347 | JN408369 | C | INDIA | 2007 | Mumbai |
| 348 | JN408370 | C | INDIA | 2007 | Mumbai |
| 349 | JN408371 | C | INDIA | 2007 | Mumbai |
| 350 | JN408372 | C | INDIA | 2007 | Mumbai |
| 351 | JN408373 | C | INDIA | 2007 | Mumbai |
| 352 | JN408374 | C | INDIA | 2007 | Mumbai |
| 353 | JN408375 | C | INDIA | 2007 | Mumbai |
| 354 | JN408376 | C | INDIA | 2007 | Mumbai |
| 355 | JN408377 | C | INDIA | 2007 | Mumbai |
| 356 | JN408378 | C | INDIA | 2007 | Mumbai |
| 357 | JN408379 | C | INDIA | 2007 | Mumbai |
| 358 | JN408380 | C | INDIA | 2007 | Mumbai |
| 359 | JN408381 | C | INDIA | 2007 | Mumbai |
| 360 | JN408382 | C | INDIA | 2007 | Mumbai |
| 361 | JN408383 | C | INDIA | 2007 | Mumbai |
| 362 | JN408384 | C | INDIA | 2007 | Mumbai |
| 363 | JN408385 | C | INDIA | 2007 | Mumbai |
| 364 | JN408386 | C | INDIA | 2007 | Mumbai |
| 365 | JN408387 | C | INDIA | 2007 | Mumbai |
| 366 | JN408388 | C | INDIA | 2007 | Mumbai |
| 367 | JN408389 | C | INDIA | 2007 | Mumbai |
| 368 | JN408390 | C | INDIA | 2007 | Mumbai |
| 369 | JN408391 | C | INDIA | 2007 | Mumbai |
| 370 | JN408392 | C | INDIA | 2007 | Mumbai |
| 371 | JN408394 | C | INDIA | 2007 | Mumbai |
| 372 | JN408395 | C | INDIA | 2007 | Mumbai |
| 373 | JN408396 | C | INDIA | 2007 | Mumbai |
| 374 | JN408397 | C | INDIA | 2007 | Mumbai |
| 375 | JN408398 | C | INDIA | 2007 | Mumbai |
| 376 | JN408399 | C | INDIA | 2007 | Mumbai |
| 377 | JN408400 | C | INDIA | 2007 | Mumbai |
| 378 | JN408401 | C | INDIA | 2007 | Mumbai |
| 379 | JN408402 | C | INDIA | 2007 | Mumbai |
| 380 | JN408403 | C | INDIA | 2007 | Mumbai |
| 381 | JN408404 | C | INDIA | 2007 | Mumbai |
| 382 | JN408405 | C | INDIA | 2007 | Mumbai |
| 383 | JN408406 | C | INDIA | 2007 | Mumbai |
| 384 | JN408407 | C | INDIA | 2007 | Mumbai |
| 385 | JN408408 | C | INDIA | 2007 | Mumbai |
| 386 | JN408409 | C | INDIA | 2007 | Mumbai |
| 387 | JN408410 | C | INDIA | 2007 | Mumbai |
| 388 | JN408411 | C | INDIA | 2007 | Mumbai |
| 389 | JN408412 | C | INDIA | 2007 | Mumbai |
| 390 | JN408413 | C | INDIA | 2007 | Mumbai |
| 391 | JN408414 | C | INDIA | 2007 | Mumbai |
| 392 | JN408415 | C | INDIA | 2007 | Mumbai |
| 393 | JN408416 | C | INDIA | 2007 | Mumbai |
| 394 | JN408417 | C | INDIA | 2007 | Mumbai |
| 395 | JN408418 | C | INDIA | 2007 | Mumbai |
| 396 | JN408419 | C | INDIA | 2007 | Mumbai |
| 397 | JN408420 | C | INDIA | 2007 | Mumbai |
| 398 | JN408421 | C | INDIA | 2007 | Mumbai |
| 399 | JN408422 | C | INDIA | 2007 | Mumbai |
| 400 | JN408423 | C | INDIA | 2007 | Mumbai |
| 401 | JN408424 | C | INDIA | 2007 | Mumbai |
| 402 | JN408425 | C | INDIA | 2007 | Mumbai |
| 403 | JN408426 | C | INDIA | 2007 | Mumbai |
| 404 | JN408427 | C | INDIA | 2007 | Mumbai |
| 405 | JN408428 | C | INDIA | 2007 | Mumbai |
| 406 | JN408429 | C | INDIA | 2007 | Mumbai |
| 407 | JN408430 | C | INDIA | 2007 | Mumbai |
| 408 | JN408431 | C | INDIA | 2007 | Mumbai |
| 409 | JN408432 | C | INDIA | 2007 | Mumbai |
| 410 | JN408433 | C | INDIA | 2007 | Mumbai |
| 411 | JN408434 | C | INDIA | 2007 | Mumbai |
| 412 | JN408435 | C | INDIA | 2007 | Mumbai |
| 413 | JN408436 | C | INDIA | 2007 | Mumbai |
| 414 | JN408437 | C | INDIA | 2007 | Mumbai |
| 415 | JN408438 | C | INDIA | 2007 | Mumbai |
| 416 | JN408439 | C | INDIA | 2007 | Mumbai |
| 417 | JN408440 | C | INDIA | 2007 | Mumbai |
| 418 | JN408441 | C | INDIA | 2007 | Mumbai |
| 419 | JN408442 | C | INDIA | 2007 | Mumbai |
| 420 | JN408443 | C | INDIA | 2007 | Mumbai |
| 421 | JN408444 | C | INDIA | 2007 | Mumbai |
| 422 | JN408445 | C | INDIA | 2007 | Mumbai |
| 423 | JN408446 | C | INDIA | 2007 | Mumbai |
| 424 | JN408447 | C | INDIA | 2007 | Mumbai |
| 425 | JN408448 | C | INDIA | 2007 | Mumbai |
| 426 | JN634317 | C | INDIA | 2007 | Not Available |
| 427 | JN634433 | C | INDIA | 2007 | Not Available |
| 428 | JN634434 | C | INDIA | 2007 | Not Available |
| 429 | JN634435 | C | INDIA | 2007 | Not Available |
| 430 | KT833401 | C | INDIA | 2007 | Not Available |
| 431 | KT833402 | C | INDIA | 2007 | Not Available |
| 432 | KT833403 | C | INDIA | 2007 | Not Available |
| 433 | MF573228 | C | INDIA | 2007 | Not Available |
| 434 | MF573244 | C | INDIA | 2007 | Not Available |
| 435 | MF594860 | C | INDIA | 2007 | Not Available |
| 436 | EU683794 | C | INDIA | 2008 | Not Available |
| 437 | EU781841 | C | INDIA | 2008 | Not Available |
| 438 | EU781842 | C | INDIA | 2008 | Not Available |
| 439 | EU781844 | C | INDIA | 2008 | Not Available |
| 440 | HQ166846 | C | INDIA | 2008 | KAKINADA |
| 441 | HQ166847 | C | INDIA | 2008 | KAKINADA |
| 442 | HQ166848 | C | INDIA | 2008 | KAKINADA |
| 443 | HQ166849 | C | INDIA | 2008 | KAKINADA |
| 444 | HQ166850 | C | INDIA | 2008 | KAKINADA |
| 445 | HQ166851 | C | INDIA | 2008 | KAKINADA |
| 446 | HQ166852 | C | INDIA | 2008 | KAKINADA |
| 447 | HQ166853 | C | INDIA | 2008 | KAKINADA |
| 448 | HQ166854 | C | INDIA | 2008 | KAKINADA |
| 449 | HQ166855 | C | INDIA | 2008 | KAKINADA |
| 450 | HQ166856 | C | INDIA | 2008 | KAKINADA |
| 451 | HQ166857 | C | INDIA | 2008 | KAKINADA |
| 452 | HQ166858 | C | INDIA | 2008 | KAKINADA |
| 453 | HQ166859 | C | INDIA | 2008 | KAKINADA |
| 454 | HQ166860 | C | INDIA | 2008 | KAKINADA |
| 455 | HQ166861 | C | INDIA | 2008 | KAKINADA |
| 456 | HQ166862 | C | INDIA | 2008 | KAKINADA |
| 457 | HQ166863 | C | INDIA | 2008 | KAKINADA |
| 458 | HQ166864 | C | INDIA | 2008 | KAKINADA |
| 459 | HQ166865 | C | INDIA | 2008 | KAKINADA |
| 460 | HQ166866 | C | INDIA | 2008 | KAKINADA |
| 461 | HQ166867 | C | INDIA | 2008 | KAKINADA |
| 462 | HQ166868 | C | INDIA | 2008 | KAKINADA |
| 463 | HQ166869 | C | INDIA | 2008 | KAKINADA |
| 464 | HQ166870 | C | INDIA | 2008 | KAKINADA |
| 465 | HQ166871 | C | INDIA | 2008 | KAKINADA |
| 466 | HQ166872 | C | INDIA | 2008 | KAKINADA |
| 467 | HQ166873 | C | INDIA | 2008 | KAKINADA |
| 468 | HQ166874 | C | INDIA | 2008 | KAKINADA |
| 469 | HQ166875 | C | INDIA | 2008 | KAKINADA |
| 470 | HQ166876 | C | INDIA | 2008 | KAKINADA |
| 471 | HQ166877 | C | INDIA | 2008 | KAKINADA |
| 472 | HQ166878 | C | INDIA | 2008 | KAKINADA |
| 473 | HQ166879 | C | INDIA | 2008 | KAKINADA |
| 474 | HQ166880 | C | INDIA | 2008 | KAKINADA |
| 475 | HQ166881 | C | INDIA | 2008 | KAKINADA |
| 476 | HQ166882 | C | INDIA | 2008 | KAKINADA |
| 477 | HQ166883 | C | INDIA | 2008 | KAKINADA |
| 478 | HQ166884 | C | INDIA | 2008 | KAKINADA |
| 479 | HQ166885 | C | INDIA | 2008 | KAKINADA |
| 480 | HQ166886 | C | INDIA | 2008 | KAKINADA |
| 481 | HQ166887 | C | INDIA | 2008 | KAKINADA |
| 482 | HQ453369 | C | INDIA | 2008 | DELHI |
| 483 | HQ453377 | C | INDIA | 2008 | DELHI |
| 484 | HQ453378 | C | INDIA | 2008 | DELHI |
| 485 | HQ453382 | C | INDIA | 2008 | DELHI |
| 486 | HQ453385 | C | INDIA | 2008 | DELHI |
| 487 | HQ453392 | C | INDIA | 2008 | DELHI |
| 488 | HQ453393 | C | INDIA | 2008 | DELHI |
| 489 | HQ453394 | C | INDIA | 2008 | DELHI |
| 490 | HQ456667 | C | INDIA | 2008 | Not Available |
| 491 | HQ456668 | C | INDIA | 2008 | Not Available |
| 492 | HQ456669 | C | INDIA | 2008 | Not Available |
| 493 | HQ456671 | C | INDIA | 2008 | Not Available |
| 494 | JN314892 | C | INDIA | 2008 | Chennai |
| 495 | JN314893 | C | INDIA | 2008 | Chennai |
| 496 | JN314894 | C | INDIA | 2008 | Chennai |
| 497 | JN314895 | C | INDIA | 2008 | Chennai |
| 498 | JN314896 | C | INDIA | 2008 | Chennai |
| 499 | JN314897 | C | INDIA | 2008 | Chennai |
| 500 | JN314898 | C | INDIA | 2008 | Chennai |
| 501 | JN314899 | C | INDIA | 2008 | Chennai |
| 502 | JN314900 | C | INDIA | 2008 | Chennai |
| 503 | JN314901 | C | INDIA | 2008 | Chennai |
| 504 | JN314902 | C | INDIA | 2008 | Chennai |
| 505 | JN314903 | C | INDIA | 2008 | Chennai |
| 506 | JN314904 | C | INDIA | 2008 | Chennai |
| 507 | JN314905 | C | INDIA | 2008 | Chennai |
| 508 | JN314906 | C | INDIA | 2008 | Chennai |
| 509 | JN314907 | C | INDIA | 2008 | Chennai |
| 510 | JN314908 | C | INDIA | 2008 | Chennai |
| 511 | JN314909 | C | INDIA | 2008 | Chennai |
| 512 | JN314910 | C | INDIA | 2008 | Chennai |
| 513 | JN314911 | C | INDIA | 2008 | Chennai |
| 514 | JN314912 | C | INDIA | 2008 | Chennai |
| 515 | JN314913 | C | INDIA | 2008 | Chennai |
| 516 | JN314914 | C | INDIA | 2008 | Chennai |
| 517 | JN314915 | C | INDIA | 2008 | Chennai |
| 518 | JN314916 | C | INDIA | 2008 | Chennai |
| 519 | JN314917 | C | INDIA | 2008 | Chennai |
| 520 | JN314918 | C | INDIA | 2008 | Chennai |
| 521 | JN314919 | C | INDIA | 2008 | Chennai |
| 522 | JN314920 | C | INDIA | 2008 | Chennai |
| 523 | JN314921 | C | INDIA | 2008 | Chennai |
| 524 | JN314922 | C | INDIA | 2008 | Chennai |
| 525 | JN314923 | C | INDIA | 2008 | Chennai |
| 526 | JN314924 | C | INDIA | 2008 | Chennai |
| 527 | JN314925 | C | INDIA | 2008 | Chennai |
| 528 | JN314926 | C | INDIA | 2008 | Chennai |
| 529 | JN314927 | C | INDIA | 2008 | Chennai |
| 530 | JN314928 | C | INDIA | 2008 | Chennai |
| 531 | JN314929 | C | INDIA | 2008 | Chennai |
| 532 | JN314930 | C | INDIA | 2008 | Chennai |
| 533 | JN314931 | C | INDIA | 2008 | Chennai |
| 534 | JN314932 | C | INDIA | 2008 | Chennai |
| 535 | JN314933 | C | INDIA | 2008 | Chennai |
| 536 | JN314934 | C | INDIA | 2008 | Chennai |
| 537 | JN314935 | C | INDIA | 2008 | Chennai |
| 538 | JN314936 | C | INDIA | 2008 | Chennai |
| 539 | JN314937 | C | INDIA | 2008 | Chennai |
| 540 | JN314938 | C | INDIA | 2008 | Chennai |
| 541 | JN314939 | C | INDIA | 2008 | Chennai |
| 542 | JN314940 | C | INDIA | 2008 | Chennai |
| 543 | JN314941 | C | INDIA | 2008 | Chennai |
| 544 | JN314942 | C | INDIA | 2008 | Chennai |
| 545 | JN314943 | C | INDIA | 2008 | Chennai |
| 546 | JN314944 | C | INDIA | 2008 | Chennai |
| 547 | JN314945 | C | INDIA | 2008 | Chennai |
| 548 | JN314946 | C | INDIA | 2008 | Chennai |
| 549 | JN314947 | C | INDIA | 2008 | Chennai |
| 550 | JN314948 | C | INDIA | 2008 | Chennai |
| 551 | JN314949 | C | INDIA | 2008 | Chennai |
| 552 | JN314950 | C | INDIA | 2008 | Chennai |
| 553 | JN314951 | C | INDIA | 2008 | Chennai |
| 554 | JN314952 | C | INDIA | 2008 | Chennai |
| 555 | JN314953 | C | INDIA | 2008 | Chennai |
| 556 | JN314954 | C | INDIA | 2008 | Chennai |
| 557 | JN314955 | C | INDIA | 2008 | Chennai |
| 558 | JN314956 | C | INDIA | 2008 | Chennai |
| 559 | JN314957 | C | INDIA | 2008 | Chennai |
| 560 | JN314958 | C | INDIA | 2008 | Chennai |
| 561 | JN314959 | C | INDIA | 2008 | Chennai |
| 562 | JN314960 | C | INDIA | 2008 | Chennai |
| 563 | JN314961 | C | INDIA | 2008 | Chennai |
| 564 | JN314962 | C | INDIA | 2008 | Chennai |
| 565 | JN314963 | C | INDIA | 2008 | Chennai |
| 566 | JN314964 | C | INDIA | 2008 | Chennai |
| 567 | JN314965 | C | INDIA | 2008 | Chennai |
| 568 | JN314966 | C | INDIA | 2008 | Chennai |
| 569 | JN314967 | C | INDIA | 2008 | Chennai |
| 570 | JN314968 | C | INDIA | 2008 | Chennai |
| 571 | JN314969 | C | INDIA | 2008 | Chennai |
| 572 | JN314970 | C | INDIA | 2008 | Chennai |
| 573 | JN314971 | C | INDIA | 2008 | Chennai |
| 574 | JN314972 | C | INDIA | 2008 | Chennai |
| 575 | JN314973 | C | INDIA | 2008 | Chennai |
| 576 | JN314974 | C | INDIA | 2008 | Chennai |
| 577 | JN314975 | C | INDIA | 2008 | Chennai |
| 578 | JN314976 | C | INDIA | 2008 | Chennai |
| 579 | JN314977 | C | INDIA | 2008 | Chennai |
| 580 | JN314978 | C | INDIA | 2008 | Chennai |
| 581 | JN314979 | C | INDIA | 2008 | Chennai |
| 582 | JN314980 | C | INDIA | 2008 | Chennai |
| 583 | JN314981 | C | INDIA | 2008 | Chennai |
| 584 | JN314982 | C | INDIA | 2008 | Chennai |
| 585 | JN314983 | C | INDIA | 2008 | Chennai |
| 586 | JN314984 | C | INDIA | 2008 | Chennai |
| 587 | JN314985 | C | INDIA | 2008 | Chennai |
| 588 | JN314986 | C | INDIA | 2008 | Chennai |
| 589 | JN314987 | C | INDIA | 2008 | Chennai |
| 590 | JN634436 | C | INDIA | 2008 | Not Available |
| 591 | JN634437 | C | INDIA | 2008 | Not Available |
| 592 | JN634438 | C | INDIA | 2008 | Not Available |
| 593 | JN634439 | C | INDIA | 2008 | Not Available |
| 594 | KT833404 | C | INDIA | 2008 | Not Available |
| 595 | KT833405 | C | INDIA | 2008 | Not Available |
| 596 | KT833406 | C | INDIA | 2008 | Not Available |
| 597 | KT833407 | C | INDIA | 2008 | Not Available |
| 598 | KT833408 | C | INDIA | 2008 | Not Available |
| 599 | KT833409 | C | INDIA | 2008 | Not Available |
| 600 | KT833410 | C | INDIA | 2008 | Not Available |
| 601 | KT833422 | C | INDIA | 2008 | Not Available |
| 602 | KT833423 | C | INDIA | 2008 | Not Available |
| 603 | KT833425 | C | INDIA | 2008 | Not Available |
| 604 | KT833428 | C | INDIA | 2008 | Not Available |
| 605 | KU562074 | C | INDIA | 2008 | Not Available |
| 606 | KU562076 | C | INDIA | 2008 | Not Available |
| 607 | MF573234 | C | INDIA | 2008 | Not Available |
| 608 | MF594833 | C | INDIA | 2008 | Not Available |
| 609 | HQ456670 | C | INDIA | 2009 | Not Available |
| 610 | HQ456672 | C | INDIA | 2009 | Not Available |
| 611 | HQ456673 | C | INDIA | 2009 | Not Available |
| 612 | HQ456674 | C | INDIA | 2009 | Not Available |
| 613 | HQ456678 | C | INDIA | 2009 | Not Available |
| 614 | HQ456679 | C | INDIA | 2009 | Not Available |
| 615 | HQ456680 | C | INDIA | 2009 | Not Available |
| 616 | HQ456682 | C | INDIA | 2009 | Not Available |
| 617 | JN634440 | C | INDIA | 2009 | Not Available |
| 618 | JN634441 | C | INDIA | 2009 | Not Available |
| 619 | JN634442 | C | INDIA | 2009 | Not Available |
| 620 | JN639188 | C | INDIA | 2009 | Pune |
| 621 | JN639189 | C | INDIA | 2009 | Pune |
| 622 | JN639190 | C | INDIA | 2009 | Pune |
| 623 | JN639191 | C | INDIA | 2009 | Pune |
| 624 | JN639192 | C | INDIA | 2009 | Pune |
| 625 | JN639193 | C | INDIA | 2009 | Pune |
| 626 | JN639194 | C | INDIA | 2009 | Pune |
| 627 | JN639195 | C | INDIA | 2009 | Pune |
| 628 | JN639196 | C | INDIA | 2009 | Pune |
| 629 | JN639197 | C | INDIA | 2009 | Pune |
| 630 | JN639199 | C | INDIA | 2009 | Pune |
| 631 | KC156179 | C | INDIA | 2009 | Not Available |
| 632 | KT833411 | C | INDIA | 2009 | Not Available |
| 633 | KT833412 | C | INDIA | 2009 | Not Available |
| 634 | KT833413 | C | INDIA | 2009 | Not Available |
| 635 | KT833414 | C | INDIA | 2009 | Not Available |
| 636 | KT833415 | C | INDIA | 2009 | Not Available |
| 637 | KT833416 | C | INDIA | 2009 | Not Available |
| 638 | KT833417 | C | INDIA | 2009 | Not Available |
| 639 | KT833418 | C | INDIA | 2009 | Not Available |
| 640 | KT833419 | C | INDIA | 2009 | Not Available |
| 641 | KT833420 | C | INDIA | 2009 | Not Available |
| 642 | KT833421 | C | INDIA | 2009 | Not Available |
| 643 | KT833424 | C | INDIA | 2009 | Not Available |
| 644 | KT833426 | C | INDIA | 2009 | Not Available |
| 645 | KT833427 | C | INDIA | 2009 | Not Available |
| 646 | HQ456675 | C | INDIA | 2010 | Not Available |
| 647 | HQ456676 | C | INDIA | 2010 | Not Available |
| 648 | JN639200 | C | INDIA | 2010 | Pune |
| 649 | JN639201 | C | INDIA | 2010 | Pune |
| 650 | JN639202 | C | INDIA | 2010 | Pune |
| 651 | JN639203 | C | INDIA | 2010 | Pune |
| 652 | JN639205 | C | INDIA | 2010 | Pune |
| 653 | JN639206 | C | INDIA | 2010 | Pune |
| 654 | JN639207 | C | INDIA | 2010 | Pune |
| 655 | JN639208 | C | INDIA | 2010 | Pune |
| 656 | JN639209 | C | INDIA | 2010 | Pune |
| 657 | JN639210 | C | INDIA | 2010 | Pune |
| 658 | JN639211 | C | INDIA | 2010 | Pune |
| 659 | JN639212 | C | INDIA | 2010 | Pune |
| 660 | JN639213 | C | INDIA | 2010 | Pune |
| 661 | JN639214 | C | INDIA | 2010 | Pune |
| 662 | KF890214 | C | INDIA | 2010 | Not Available |
| 663 | KF890215 | C | INDIA | 2010 | Not Available |
| 664 | KF890216 | C | INDIA | 2010 | Not Available |
| 665 | KF890217 | C | INDIA | 2010 | Not Available |
| 666 | KF890218 | C | INDIA | 2010 | Not Available |
| 667 | KF890219 | C | INDIA | 2010 | Not Available |
| 668 | KF890220 | C | INDIA | 2010 | Not Available |
| 669 | KF890221 | C | INDIA | 2010 | Not Available |
| 670 | KF890222 | C | INDIA | 2010 | Not Available |
| 671 | KF890223 | C | INDIA | 2010 | Not Available |
| 672 | KF890224 | C | INDIA | 2010 | Not Available |
| 673 | KF890225 | C | INDIA | 2010 | Not Available |
| 674 | KF890226 | C | INDIA | 2010 | Not Available |
| 675 | KF890227 | C | INDIA | 2010 | Not Available |
| 676 | KF890228 | C | INDIA | 2010 | Not Available |
| 677 | KF890229 | C | INDIA | 2010 | Not Available |
| 678 | KF890230 | C | INDIA | 2010 | Not Available |
| 679 | KF890231 | C | INDIA | 2010 | Not Available |
| 680 | KF890232 | C | INDIA | 2010 | Not Available |
| 681 | KF890233 | C | INDIA | 2010 | Not Available |
| 682 | KF890234 | C | INDIA | 2010 | Not Available |
| 683 | KF890235 | C | INDIA | 2010 | Not Available |
| 684 | KF890236 | C | INDIA | 2010 | Not Available |
| 685 | KF890237 | C | INDIA | 2010 | Not Available |
| 686 | KF890238 | C | INDIA | 2010 | Not Available |
| 687 | KF890239 | C | INDIA | 2010 | Not Available |
| 688 | KF890240 | C | INDIA | 2010 | Not Available |
| 689 | KF890241 | C | INDIA | 2010 | Not Available |
| 690 | KF890242 | C | INDIA | 2010 | Not Available |
| 691 | KF890243 | C | INDIA | 2010 | Not Available |
| 692 | KF890244 | C | INDIA | 2010 | Not Available |
| 693 | KF890245 | C | INDIA | 2010 | Not Available |
| 694 | KF890246 | C | INDIA | 2010 | Not Available |
| 695 | KF890247 | C | INDIA | 2010 | Not Available |
| 696 | KF890248 | C | INDIA | 2010 | Not Available |
| 697 | KF890249 | C | INDIA | 2010 | Not Available |
| 698 | KF890250 | C | INDIA | 2010 | Not Available |
| 699 | KF890251 | C | INDIA | 2010 | Not Available |
| 700 | MF594830 | C | INDIA | 2010 | Not Available |
| 701 | KX069227 | C | INDIA | 2011 | Chennai |
| 702 | KX069228 | C | INDIA | 2011 | Chennai |
| 703 | MF573236 | C | INDIA | 2011 | Not Available |
| 704 | MF573238 | C | INDIA | 2011 | Not Available |
| 705 | MF573242 | C | INDIA | 2011 | Not Available |
| 706 | MF594834 | C | INDIA | 2011 | Not Available |
| 707 | MF594838 | C | INDIA | 2011 | Not Available |
| 708 | MF594844 | C | INDIA | 2011 | Not Available |
| 709 | MF594846 | C | INDIA | 2011 | Not Available |
| 710 | MF594856 | C | INDIA | 2011 | Not Available |
| 711 | MF594858 | C | INDIA | 2011 | Not Available |
| 712 | MK330780 | C | INDIA | 2011 | SOUTH INDIA |
| 713 | MK330782 | C | INDIA | 2011 | SOUTH INDIA |
| 714 | MK330783 | C | INDIA | 2011 | SOUTH INDIA |
| 715 | MK330784 | C | INDIA | 2011 | SOUTH INDIA |
| 716 | MK330785 | C | INDIA | 2011 | SOUTH INDIA |
| 717 | MK330786 | C | INDIA | 2011 | SOUTH INDIA |
| 718 | MK330787 | C | INDIA | 2011 | SOUTH INDIA |
| 719 | MK330790 | C | INDIA | 2011 | SOUTH INDIA |
| 720 | MK330792 | C | INDIA | 2011 | SOUTH INDIA |
| 721 | MK330793 | C | INDIA | 2011 | SOUTH INDIA |
| 722 | MK330795 | C | INDIA | 2011 | SOUTH INDIA |
| 723 | MK330796 | C | INDIA | 2011 | SOUTH INDIA |
| 724 | MK330797 | C | INDIA | 2011 | SOUTH INDIA |
| 725 | MK330798 | C | INDIA | 2011 | SOUTH INDIA |
| 726 | MK330799 | C | INDIA | 2011 | SOUTH INDIA |
| 727 | MK330800 | C | INDIA | 2011 | SOUTH INDIA |
| 728 | MK330801 | C | INDIA | 2011 | SOUTH INDIA |
| 729 | MK330802 | C | INDIA | 2011 | SOUTH INDIA |
| 730 | MK330803 | C | INDIA | 2011 | SOUTH INDIA |
| 731 | MK330804 | C | INDIA | 2011 | SOUTH INDIA |
| 732 | MK330805 | C | INDIA | 2011 | SOUTH INDIA |
| 733 | MK330806 | C | INDIA | 2011 | SOUTH INDIA |
| 734 | MK330807 | C | INDIA | 2011 | SOUTH INDIA |
| 735 | MK330808 | C | INDIA | 2011 | SOUTH INDIA |
| 736 | MK330809 | C | INDIA | 2011 | SOUTH INDIA |
| 737 | MK330810 | C | INDIA | 2011 | SOUTH INDIA |
| 738 | MK330812 | C | INDIA | 2011 | SOUTH INDIA |
| 739 | MK330813 | C | INDIA | 2011 | SOUTH INDIA |
| 740 | MK330814 | C | INDIA | 2011 | SOUTH INDIA |
| 741 | MK330815 | C | INDIA | 2011 | SOUTH INDIA |
| 742 | MK330816 | C | INDIA | 2011 | SOUTH INDIA |
| 743 | MK330817 | C | INDIA | 2011 | SOUTH INDIA |
| 744 | MK330818 | C | INDIA | 2011 | SOUTH INDIA |
| 745 | MK330819 | C | INDIA | 2011 | SOUTH INDIA |
| 746 | MK330820 | C | INDIA | 2011 | SOUTH INDIA |
| 747 | MK330822 | C | INDIA | 2011 | SOUTH INDIA |
| 748 | MK330823 | C | INDIA | 2011 | SOUTH INDIA |
| 749 | MK330824 | C | INDIA | 2011 | SOUTH INDIA |
| 750 | MK330825 | C | INDIA | 2011 | SOUTH INDIA |
| 751 | MK330827 | C | INDIA | 2011 | SOUTH INDIA |
| 752 | MK330828 | C | INDIA | 2011 | SOUTH INDIA |
| 753 | MK330829 | C | INDIA | 2011 | SOUTH INDIA |
| 754 | MK639604 | C | INDIA | 2011 | SOUTH INDIA |
| 755 | KJ185171 | C | INDIA | 2012 | Not Available |
| 756 | KJ185172 | C | INDIA | 2012 | Not Available |
| 757 | KJ185173 | C | INDIA | 2012 | Not Available |
| 758 | KJ185174 | C | INDIA | 2012 | Not Available |
| 759 | KJ185175 | C | INDIA | 2012 | Not Available |
| 760 | KJ185176 | C | INDIA | 2012 | Not Available |
| 761 | KJ185177 | C | INDIA | 2012 | Not Available |
| 762 | KJ185178 | C | INDIA | 2012 | Not Available |
| 763 | KJ185179 | C | INDIA | 2012 | Not Available |
| 764 | KJ185180 | C | INDIA | 2012 | Not Available |
| 765 | KJ185181 | C | INDIA | 2012 | Not Available |
| 766 | KJ185182 | C | INDIA | 2012 | Not Available |
| 767 | KJ185183 | C | INDIA | 2012 | Not Available |
| 768 | KJ185184 | C | INDIA | 2012 | Not Available |
| 769 | KJ185185 | C | INDIA | 2012 | Not Available |
| 770 | KJ185186 | C | INDIA | 2012 | Not Available |
| 771 | KJ185187 | C | INDIA | 2012 | Not Available |
| 772 | KJ185188 | C | INDIA | 2012 | Not Available |
| 773 | KJ185189 | C | INDIA | 2012 | Not Available |
| 774 | KJ185190 | C | INDIA | 2012 | Not Available |
| 775 | KJ185191 | C | INDIA | 2012 | Not Available |
| 776 | KJ185192 | C | INDIA | 2012 | Not Available |
| 777 | KJ185193 | C | INDIA | 2012 | Not Available |
| 778 | KJ185194 | C | INDIA | 2012 | Not Available |
| 779 | KJ185195 | C | INDIA | 2012 | Not Available |
| 780 | KJ185196 | C | INDIA | 2012 | Not Available |
| 781 | KJ185197 | C | INDIA | 2012 | Not Available |
| 782 | KJ185198 | C | INDIA | 2012 | Not Available |
| 783 | KJ185201 | C | INDIA | 2012 | Not Available |
| 784 | KJ185202 | C | INDIA | 2012 | Not Available |
| 785 | KJ185203 | C | INDIA | 2012 | Not Available |
| 786 | KJ185204 | C | INDIA | 2012 | Not Available |
| 787 | KJ185206 | C | INDIA | 2012 | Not Available |
| 788 | KJ185207 | C | INDIA | 2012 | Not Available |
| 789 | KJ185208 | C | INDIA | 2012 | Not Available |
| 790 | KJ185209 | C | INDIA | 2012 | Not Available |
| 791 | KJ185210 | C | INDIA | 2012 | Not Available |
| 792 | KJ185211 | C | INDIA | 2012 | Not Available |
| 793 | KJ185212 | C | INDIA | 2012 | Not Available |
| 794 | KJ185213 | C | INDIA | 2012 | Not Available |
| 795 | KJ185214 | C | INDIA | 2012 | Not Available |
| 796 | KJ185215 | C | INDIA | 2012 | Not Available |
| 797 | KJ185216 | C | INDIA | 2012 | Not Available |
| 798 | KJ185217 | C | INDIA | 2012 | Not Available |
| 799 | KJ185218 | C | INDIA | 2012 | Not Available |
| 800 | KJ185220 | C | INDIA | 2012 | Not Available |
| 801 | KJ185221 | C | INDIA | 2012 | Not Available |
| 802 | KJ185222 | C | INDIA | 2012 | Not Available |
| 803 | KJ185224 | C | INDIA | 2012 | Not Available |
| 804 | KJ185225 | C | INDIA | 2012 | Not Available |
| 805 | KJ185226 | C | INDIA | 2012 | Not Available |
| 806 | KJ185227 | C | INDIA | 2012 | Not Available |
| 807 | KJ185229 | C | INDIA | 2012 | Not Available |
| 808 | KJ185230 | C | INDIA | 2012 | Not Available |
| 809 | KJ185231 | C | INDIA | 2012 | Not Available |
| 810 | KJ185232 | C | INDIA | 2012 | Not Available |
| 811 | KJ185234 | C | INDIA | 2012 | Not Available |
| 812 | KJ185235 | C | INDIA | 2012 | Not Available |
| 813 | KJ185236 | C | INDIA | 2012 | Not Available |
| 814 | KJ185237 | C | INDIA | 2012 | Not Available |
| 815 | KJ185238 | C | INDIA | 2012 | Not Available |
| 816 | KJ185239 | C | INDIA | 2012 | Not Available |
| 817 | KJ185240 | C | INDIA | 2012 | Not Available |
| 818 | KJ185241 | C | INDIA | 2012 | Not Available |
| 819 | KJ185242 | C | INDIA | 2012 | Not Available |
| 820 | KJ185243 | C | INDIA | 2012 | Not Available |
| 821 | KJ185244 | C | INDIA | 2012 | Not Available |
| 822 | KJ185246 | C | INDIA | 2012 | Not Available |
| 823 | KJ185247 | C | INDIA | 2012 | Not Available |
| 824 | KJ185248 | C | INDIA | 2012 | Not Available |
| 825 | KJ185249 | C | INDIA | 2012 | Not Available |
| 826 | KJ185250 | C | INDIA | 2012 | Not Available |
| 827 | KJ185251 | C | INDIA | 2012 | Not Available |
| 828 | KJ185252 | C | INDIA | 2012 | Not Available |
| 829 | KJ185253 | C | INDIA | 2012 | Not Available |
| 830 | KJ185254 | C | INDIA | 2012 | Not Available |
| 831 | KJ185255 | C | INDIA | 2012 | Not Available |
| 832 | KJ185259 | C | INDIA | 2012 | Not Available |
| 833 | KJ185261 | C | INDIA | 2012 | Not Available |
| 834 | KJ185262 | C | INDIA | 2012 | Not Available |
| 835 | KJ185263 | C | INDIA | 2012 | Not Available |
| 836 | KJ185264 | C | INDIA | 2012 | Not Available |
| 837 | KM276704 | C | INDIA | 2012 | Not Available |
| 838 | MF573232 | C | INDIA | 2012 | Not Available |
| 839 | MF573240 | C | INDIA | 2012 | Not Available |
| 840 | MF594831 | C | INDIA | 2012 | Not Available |
| 841 | MF594836 | C | INDIA | 2012 | Not Available |
| 842 | MF594840 | C | INDIA | 2012 | Not Available |
| 843 | MF594850 | C | INDIA | 2012 | Not Available |
| 844 | MF594854 | C | INDIA | 2012 | Not Available |
| 845 | MK330830 | C | INDIA | 2012 | SOUTH INDIA |
| 846 | KJ185265 | C | INDIA | 2013 | Not Available |
| 847 | KJ185267 | C | INDIA | 2013 | Not Available |
| 848 | KJ185269 | C | INDIA | 2013 | Not Available |
| 849 | KJ185271 | C | INDIA | 2013 | Not Available |
| 850 | KJ185273 | C | INDIA | 2013 | Not Available |
| 851 | KJ185274 | C | INDIA | 2013 | Not Available |
| 852 | KJ185275 | C | INDIA | 2013 | Not Available |
| 853 | KJ185276 | C | INDIA | 2013 | Not Available |
| 854 | KJ185278 | C | INDIA | 2013 | Not Available |
| 855 | KJ185279 | C | INDIA | 2013 | Not Available |
| 856 | KJ185280 | C | INDIA | 2013 | Not Available |
| 857 | KJ185281 | C | INDIA | 2013 | Not Available |
| 858 | KJ185283 | C | INDIA | 2013 | Not Available |
| 859 | KJ185284 | C | INDIA | 2013 | Not Available |
| 860 | KJ185285 | C | INDIA | 2013 | Not Available |
| 861 | KJ185286 | C | INDIA | 2013 | Not Available |
| 862 | KJ185287 | C | INDIA | 2013 | Not Available |
| 863 | KJ185288 | C | INDIA | 2013 | Not Available |
| 864 | KJ185289 | C | INDIA | 2013 | Not Available |
| 865 | KJ185290 | C | INDIA | 2013 | Not Available |
| 866 | KJ185291 | C | INDIA | 2013 | Not Available |
| 867 | KJ185293 | C | INDIA | 2013 | Not Available |
| 868 | KJ185295 | C | INDIA | 2013 | Not Available |
| 869 | KJ185296 | C | INDIA | 2013 | Not Available |
| 870 | KJ185297 | C | INDIA | 2013 | Not Available |
| 871 | KJ185298 | C | INDIA | 2013 | Not Available |
| 872 | KJ185299 | C | INDIA | 2013 | Not Available |
| 873 | KJ185300 | C | INDIA | 2013 | Not Available |
| 874 | KJ185302 | C | INDIA | 2013 | Not Available |
| 875 | KJ185303 | C | INDIA | 2013 | Not Available |
| 876 | KJ185304 | C | INDIA | 2013 | Not Available |
| 877 | KJ185305 | C | INDIA | 2013 | Not Available |
| 878 | KJ185306 | C | INDIA | 2013 | Not Available |
| 879 | KJ185307 | C | INDIA | 2013 | Not Available |
| 880 | KJ185308 | C | INDIA | 2013 | Not Available |
| 881 | KJ185309 | C | INDIA | 2013 | Not Available |
| 882 | KJ185310 | C | INDIA | 2013 | Not Available |
| 883 | KJ185311 | C | INDIA | 2013 | Not Available |
| 884 | KJ185312 | C | INDIA | 2013 | Not Available |
| 885 | KJ185313 | C | INDIA | 2013 | Not Available |
| 886 | KJ185314 | C | INDIA | 2013 | Not Available |
| 887 | KJ185315 | C | INDIA | 2013 | Not Available |
| 888 | KJ185318 | C | INDIA | 2013 | Not Available |
| 889 | KJ185319 | C | INDIA | 2013 | Not Available |
| 890 | KJ185321 | C | INDIA | 2013 | Not Available |
| 891 | KJ185323 | C | INDIA | 2013 | Not Available |
| 892 | KJ185324 | C | INDIA | 2013 | Not Available |
| 893 | KJ185325 | C | INDIA | 2013 | Not Available |
| 894 | KJ185328 | C | INDIA | 2013 | Not Available |
| 895 | KJ185329 | C | INDIA | 2013 | Not Available |
| 896 | KJ185330 | C | INDIA | 2013 | Not Available |
| 897 | KJ185331 | C | INDIA | 2013 | Not Available |
| 898 | KJ185332 | C | INDIA | 2013 | Not Available |
| 899 | KJ185333 | C | INDIA | 2013 | Not Available |
| 900 | KJ185335 | C | INDIA | 2013 | Not Available |
| 901 | KJ185336 | C | INDIA | 2013 | Not Available |
| 902 | KJ185337 | C | INDIA | 2013 | Not Available |
| 903 | KJ185338 | C | INDIA | 2013 | Not Available |
| 904 | KJ185339 | C | INDIA | 2013 | Not Available |
| 905 | KJ185341 | C | INDIA | 2013 | Not Available |
| 906 | KJ185342 | C | INDIA | 2013 | Not Available |
| 907 | KJ185343 | C | INDIA | 2013 | Not Available |
| 908 | KJ185344 | C | INDIA | 2013 | Not Available |
| 909 | KJ185345 | C | INDIA | 2013 | Not Available |
| 910 | KJ185346 | C | INDIA | 2013 | Not Available |
| 911 | KJ185347 | C | INDIA | 2013 | Not Available |
| 912 | KJ185348 | C | INDIA | 2013 | Not Available |
| 913 | KJ185349 | C | INDIA | 2013 | Not Available |
| 914 | KJ185350 | C | INDIA | 2013 | Not Available |
| 915 | KJ185351 | C | INDIA | 2013 | Not Available |
| 916 | KJ185352 | C | INDIA | 2013 | Not Available |
| 917 | KJ185354 | C | INDIA | 2013 | Not Available |
| 918 | KJ185356 | C | INDIA | 2013 | Not Available |
| 919 | KJ185357 | C | INDIA | 2013 | Not Available |
| 920 | KJ185358 | C | INDIA | 2013 | Not Available |
| 921 | KJ185359 | C | INDIA | 2013 | Not Available |
| 922 | KJ185360 | C | INDIA | 2013 | Not Available |
| 923 | KJ185361 | C | INDIA | 2013 | Not Available |
| 924 | KJ185362 | C | INDIA | 2013 | Not Available |
| 925 | KJ185363 | C | INDIA | 2013 | Not Available |
| 926 | KJ185364 | C | INDIA | 2013 | Not Available |
| 927 | KJ185365 | C | INDIA | 2013 | Not Available |
| 928 | KJ185367 | C | INDIA | 2013 | Not Available |
| 929 | KJ185368 | C | INDIA | 2013 | Not Available |
| 930 | KJ185369 | C | INDIA | 2013 | Not Available |
| 931 | KJ185370 | C | INDIA | 2013 | Not Available |
| 932 | KJ185372 | C | INDIA | 2013 | Not Available |
| 933 | KJ185373 | C | INDIA | 2013 | Not Available |
| 934 | KJ185374 | C | INDIA | 2013 | Not Available |
| 935 | KJ185375 | C | INDIA | 2013 | Not Available |
| 936 | KJ185376 | C | INDIA | 2013 | Not Available |
| 937 | KJ933454 | C | INDIA | 2013 | Varanasi |
| 938 | KJ933455 | C | INDIA | 2013 | Varanasi |
| 939 | KJ933456 | C | INDIA | 2013 | Varanasi |
| 940 | KJ933457 | C | INDIA | 2013 | Varanasi |
| 941 | KJ933458 | C | INDIA | 2013 | Varanasi |
| 942 | KJ933460 | C | INDIA | 2013 | Varanasi |
| 943 | KJ933461 | C | INDIA | 2013 | Varanasi |
| 944 | KJ933462 | C | INDIA | 2013 | Varanasi |
| 945 | KJ933463 | C | INDIA | 2013 | Varanasi |
| 946 | KJ933464 | C | INDIA | 2013 | Varanasi |
| 947 | KJ933466 | C | INDIA | 2013 | Varanasi |
| 948 | KJ933467 | C | INDIA | 2013 | Varanasi |
| 949 | KJ933468 | C | INDIA | 2013 | Varanasi |
| 950 | KJ933469 | C | INDIA | 2013 | Varanasi |
| 951 | KJ933470 | C | INDIA | 2013 | Varanasi |
| 952 | KJ933471 | C | INDIA | 2013 | Varanasi |
| 953 | KM276715 | C | INDIA | 2013 | Not Available |
| 954 | MF573230 | C | INDIA | 2013 | Not Available |
| 955 | MF594828 | C | INDIA | 2013 | Not Available |
| 956 | MF594842 | C | INDIA | 2013 | Not Available |
| 957 | MF594848 | C | INDIA | 2013 | Not Available |
| 958 | MF594852 | C | INDIA | 2013 | Not Available |
| 959 | KP893639 | C | INDIA | 2014 | Not Available |
| 960 | KR816018 | C | INDIA | 2014 | Pune |
| 961 | KR816019 | C | INDIA | 2014 | Pune |
| 962 | KR816020 | C | INDIA | 2014 | Pune |
| 963 | KR816021 | C | INDIA | 2014 | Pune |
| 964 | KR816022 | C | INDIA | 2014 | Pune |
| 965 | KR816023 | C | INDIA | 2014 | Pune |
| 966 | KR816024 | C | INDIA | 2014 | Pune |
| 967 | KR816025 | C | INDIA | 2014 | Pune |
| 968 | KR816026 | C | INDIA | 2014 | Pune |
| 969 | KR816027 | C | INDIA | 2014 | Pune |
| 970 | KR816028 | C | INDIA | 2014 | Pune |
| 971 | KR816029 | C | INDIA | 2014 | Pune |
| 972 | KR816030 | C | INDIA | 2014 | Pune |
| 973 | KR816031 | C | INDIA | 2014 | Pune |
| 974 | KR816032 | C | INDIA | 2014 | Pune |
| 975 | KR816033 | C | INDIA | 2014 | Pune |
| 976 | KR816034 | C | INDIA | 2014 | Pune |
| 977 | KR816035 | C | INDIA | 2014 | Pune |
| 978 | KR816036 | C | INDIA | 2014 | Pune |
| 979 | KR816037 | C | INDIA | 2014 | Pune |
| 980 | KR816038 | C | INDIA | 2014 | Pune |
| 981 | KR816039 | C | INDIA | 2014 | Pune |
| 982 | KR816040 | C | INDIA | 2014 | Pune |
| 983 | KR816041 | C | INDIA | 2014 | Pune |
| 984 | KR816042 | C | INDIA | 2014 | Pune |
| 985 | KR816043 | C | INDIA | 2014 | Pune |
| 986 | KR816044 | C | INDIA | 2014 | Pune |
| 987 | KR816045 | C | INDIA | 2014 | Pune |
| 988 | KR816046 | C | INDIA | 2014 | Pune |
| 989 | KR816047 | C | INDIA | 2014 | Pune |
| 990 | KR816048 | C | INDIA | 2014 | Pune |
| 991 | KR816049 | C | INDIA | 2014 | Pune |
| 992 | KR816050 | C | INDIA | 2014 | Pune |
| 993 | KR816051 | C | INDIA | 2014 | Pune |
| 994 | KR816052 | C | INDIA | 2014 | Pune |
| 995 | KR816053 | C | INDIA | 2014 | Pune |
| 996 | KR816054 | C | INDIA | 2014 | Pune |
| 997 | KR816055 | C | INDIA | 2014 | Pune |
| 998 | KR816056 | C | INDIA | 2014 | Pune |
| 999 | KR816057 | C | INDIA | 2014 | Pune |
| 1000 | KR816058 | C | INDIA | 2014 | Pune |
| 1001 | KR816059 | C | INDIA | 2014 | Pune |
| 1002 | KR816060 | C | INDIA | 2014 | Pune |
| 1003 | KR816061 | C | INDIA | 2014 | Pune |
| 1004 | KR816062 | C | INDIA | 2014 | Pune |
| 1005 | KR816063 | C | INDIA | 2014 | Pune |
| 1006 | KR816064 | C | INDIA | 2014 | Pune |
| 1007 | KR816065 | C | INDIA | 2014 | Pune |
| 1008 | KR816066 | C | INDIA | 2014 | Pune |
| 1009 | KR816067 | C | INDIA | 2014 | Pune |
| 1010 | KR816068 | C | INDIA | 2014 | Pune |
| 1011 | KR816069 | C | INDIA | 2014 | Pune |
| 1012 | KR816070 | C | INDIA | 2014 | Pune |
| 1013 | KR816071 | C | INDIA | 2014 | Pune |
| 1014 | KR816072 | C | INDIA | 2014 | Pune |
| 1015 | KR816073 | C | INDIA | 2014 | Pune |
| 1016 | KR816074 | C | INDIA | 2014 | Pune |
| 1017 | KR816075 | C | INDIA | 2014 | Pune |
| 1018 | KR816076 | C | INDIA | 2014 | Pune |
| 1019 | KR816078 | C | INDIA | 2014 | Pune |
| 1020 | KR816079 | C | INDIA | 2014 | Pune |
| 1021 | KR816080 | C | INDIA | 2014 | Pune |
| 1022 | KR816081 | C | INDIA | 2014 | Pune |
| 1023 | KR816082 | C | INDIA | 2014 | Pune |
| 1024 | KR816083 | C | INDIA | 2014 | Pune |
| 1025 | KR816084 | C | INDIA | 2014 | Pune |
| 1026 | KR816085 | C | INDIA | 2014 | Pune |
| 1027 | KR816086 | C | INDIA | 2014 | Pune |
| 1028 | KR816087 | C | INDIA | 2014 | Pune |
| 1029 | KR816088 | C | INDIA | 2014 | Pune |
| 1030 | KR816089 | C | INDIA | 2014 | Pune |
| 1031 | KR816090 | C | INDIA | 2014 | Pune |
| 1032 | KR816091 | C | INDIA | 2014 | Pune |
| 1033 | KR816092 | C | INDIA | 2014 | Pune |
| 1034 | KR816093 | C | INDIA | 2014 | Pune |
| 1035 | KR816095 | C | INDIA | 2014 | Pune |
| 1036 | KR816096 | C | INDIA | 2014 | Pune |
| 1037 | KR816097 | C | INDIA | 2014 | Pune |
| 1038 | KT318883 | C | INDIA | 2014 | Pune |
| 1039 | KT318884 | C | INDIA | 2014 | Pune |
| 1040 | KT318885 | C | INDIA | 2014 | Pune |
| 1041 | KT318886 | C | INDIA | 2014 | Pune |
| 1042 | KT318887 | C | INDIA | 2014 | Pune |
| 1043 | KT318888 | C | INDIA | 2014 | Pune |
| 1044 | KT318890 | C | INDIA | 2014 | Pune |
| 1045 | KT318891 | C | INDIA | 2014 | Pune |
| 1046 | KT318892 | C | INDIA | 2014 | Pune |
| 1047 | KT318893 | C | INDIA | 2014 | Pune |
| 1048 | KT318894 | C | INDIA | 2014 | Pune |
| 1049 | KT318895 | C | INDIA | 2014 | Pune |
| 1050 | KT318896 | C | INDIA | 2014 | Pune |
| 1051 | KT318897 | C | INDIA | 2014 | Pune |
| 1052 | KT318898 | C | INDIA | 2014 | Pune |
| 1053 | KT318900 | C | INDIA | 2014 | Pune |
| 1054 | KT318901 | C | INDIA | 2014 | Pune |
| 1055 | KT318902 | C | INDIA | 2014 | Pune |
| 1056 | KT318903 | C | INDIA | 2014 | Pune |
| 1057 | KT318904 | C | INDIA | 2014 | Pune |
| 1058 | KT318905 | C | INDIA | 2014 | Pune |
| 1059 | KT318906 | C | INDIA | 2014 | Pune |
| 1060 | KT318907 | C | INDIA | 2014 | Pune |
| 1061 | KT318908 | C | INDIA | 2014 | Pune |
| 1062 | KT318909 | C | INDIA | 2014 | Pune |
| 1063 | KT318910 | C | INDIA | 2014 | Pune |
| 1064 | KT318911 | C | INDIA | 2014 | Pune |
| 1065 | KT318912 | C | INDIA | 2014 | Pune |
| 1066 | KT318913 | C | INDIA | 2014 | Pune |
| 1067 | KT318914 | C | INDIA | 2014 | Pune |
| 1068 | KT318915 | C | INDIA | 2014 | Pune |
| 1069 | KT318916 | C | INDIA | 2014 | Pune |
| 1070 | KT318917 | C | INDIA | 2014 | Pune |
| 1071 | KT318918 | C | INDIA | 2014 | Pune |
| 1072 | KT318919 | C | INDIA | 2014 | Pune |
| 1073 | KT318920 | C | INDIA | 2014 | Pune |
| 1074 | KT318922 | C | INDIA | 2014 | Pune |
| 1075 | KT318924 | C | INDIA | 2014 | Pune |
| 1076 | KT318925 | C | INDIA | 2014 | Pune |
| 1077 | KT318926 | C | INDIA | 2014 | Pune |
| 1078 | KT318929 | C | INDIA | 2014 | Pune |
| 1079 | KT318930 | C | INDIA | 2014 | Pune |
| 1080 | KT318931 | C | INDIA | 2014 | Pune |
| 1081 | KT318932 | C | INDIA | 2014 | Pune |
| 1082 | KT318934 | C | INDIA | 2014 | Pune |
| 1083 | KX069222 | C | INDIA | 2014 | Chennai |
| 1084 | KY345044 | C | INDIA | 2014 | Pune |
| 1085 | KY345045 | C | INDIA | 2014 | Pune |
| 1086 | KY345046 | C | INDIA | 2014 | Pune |
| 1087 | KY345047 | C | INDIA | 2014 | Pune |
| 1088 | KY345048 | C | INDIA | 2014 | Pune |
| 1089 | KY345049 | C | INDIA | 2014 | Pune |
| 1090 | KY345050 | C | INDIA | 2014 | Pune |
| 1091 | KY345051 | C | INDIA | 2014 | Pune |
| 1092 | KY345052 | C | INDIA | 2014 | Pune |
| 1093 | KY345053 | C | INDIA | 2014 | Pune |
| 1094 | KY345054 | C | INDIA | 2014 | Pune |
| 1095 | KT318899 | C | INDIA | 2015 | Pune |
| 1096 | KT318923 | C | INDIA | 2015 | Pune |
| 1097 | KT318927 | C | INDIA | 2015 | Pune |
| 1098 | KT318928 | C | INDIA | 2015 | Pune |
| 1099 | KT318933 | C | INDIA | 2015 | Pune |
| 1100 | KX069219 | C | INDIA | 2015 | Chennai |
| 1101 | KX069220 | C | INDIA | 2015 | Chennai |
| 1102 | KX069221 | C | INDIA | 2015 | Chennai |
| 1103 | KX069223 | C | INDIA | 2015 | Chennai |
| 1104 | KX069224 | C | INDIA | 2015 | Chennai |
| 1105 | KX069225 | C | INDIA | 2015 | Chennai |
| 1106 | KX069226 | C | INDIA | 2015 | Chennai |
| 1107 | KX570603 | C | INDIA | 2015 | Maharashtra |
| 1108 | KX570604 | C | INDIA | 2015 | Maharashtra |
| 1109 | KX570605 | C | INDIA | 2015 | Maharashtra |
| 1110 | KX570606 | C | INDIA | 2015 | Maharashtra |
| 1111 | KY713230 | C | INDIA | 2015 | Bengaluru |
| 1112 | KY713231 | C | INDIA | 2015 | Bengaluru |
| 1113 | KY713234 | C | INDIA | 2015 | Bengaluru |
| 1114 | KY713235 | C | INDIA | 2015 | Bengaluru |
| 1115 | KY713236 | C | INDIA | 2015 | Bengaluru |
| 1116 | MK041591 | C | INDIA | 2015 | Not Available |
| 1117 | MK041592 | C | INDIA | 2015 | Not Available |
| 1118 | MK041593 | C | INDIA | 2015 | Not Available |
| 1119 | MK041596 | C | INDIA | 2015 | Not Available |
| 1120 | KY787033 | C | INDIA | 2016 | Pune |
| 1121 | KY787034 | C | INDIA | 2016 | Pune |
| 1122 | KY787035 | C | INDIA | 2016 | Pune |
| 1123 | KY787036 | C | INDIA | 2016 | Pune |
| 1124 | KY787037 | C | INDIA | 2016 | Pune |
| 1125 | KY787038 | C | INDIA | 2016 | Pune |
| 1126 | KY787039 | C | INDIA | 2016 | Pune |
| 1127 | KY787040 | C | INDIA | 2016 | Pune |
| 1128 | KY787041 | C | INDIA | 2016 | Pune |
| 1129 | KY787042 | C | INDIA | 2016 | Pune |
| 1130 | KY787043 | C | INDIA | 2016 | Pune |
| 1131 | KY787044 | C | INDIA | 2016 | Pune |
| 1132 | KY787045 | C | INDIA | 2016 | Pune |
| 1133 | KY787046 | C | INDIA | 2016 | Pune |
| 1134 | KY787047 | C | INDIA | 2016 | Pune |
| 1135 | KY787048 | C | INDIA | 2016 | Pune |
| 1136 | KY787049 | C | INDIA | 2016 | Pune |
| 1137 | KY787051 | C | INDIA | 2016 | Pune |
| 1138 | KY787052 | C | INDIA | 2016 | Pune |
| 1139 | KY787053 | C | INDIA | 2016 | Pune |
| 1140 | KY787054 | C | INDIA | 2016 | Pune |
| 1141 | KY787055 | C | INDIA | 2016 | Pune |
| 1142 | KY787056 | C | INDIA | 2016 | Pune |
| 1143 | KY787057 | C | INDIA | 2016 | Pune |
| 1144 | KY787058 | C | INDIA | 2016 | Pune |
| 1145 | KY787059 | C | INDIA | 2016 | Pune |
| 1146 | KY787060 | C | INDIA | 2016 | Pune |
| 1147 | KY787061 | C | INDIA | 2016 | Pune |
| 1148 | KY787062 | C | INDIA | 2016 | Pune |
| 1149 | KY787063 | C | INDIA | 2016 | Pune |
| 1150 | KY787064 | C | INDIA | 2016 | Pune |
| 1151 | KY787066 | C | INDIA | 2016 | Pune |
| 1152 | KY787067 | C | INDIA | 2016 | Pune |
| 1153 | KY787068 | C | INDIA | 2016 | Pune |
| 1154 | KY787069 | C | INDIA | 2016 | Pune |
| 1155 | KY787070 | C | INDIA | 2016 | Pune |
| 1156 | KY787071 | C | INDIA | 2016 | Pune |
| 1157 | KY787072 | C | INDIA | 2016 | Pune |
| 1158 | KY787073 | C | INDIA | 2016 | Pune |
| 1159 | KY787074 | C | INDIA | 2016 | Pune |
| 1160 | KY787075 | C | INDIA | 2016 | Pune |
| 1161 | KY787076 | C | INDIA | 2016 | Pune |
| 1162 | KY787077 | C | INDIA | 2016 | Pune |
| 1163 | KY787078 | C | INDIA | 2016 | Pune |
| 1164 | KY787079 | C | INDIA | 2016 | Pune |
| 1165 | KY787080 | C | INDIA | 2016 | Pune |
| 1166 | KY787081 | C | INDIA | 2016 | Pune |
| 1167 | KY787082 | C | INDIA | 2016 | Pune |
| 1168 | KY787083 | C | INDIA | 2016 | Pune |
| 1169 | KY787084 | C | INDIA | 2016 | Pune |
| 1170 | KY787085 | C | INDIA | 2016 | Pune |
| 1171 | KY787086 | C | INDIA | 2016 | Pune |
| 1172 | KY787087 | C | INDIA | 2016 | Pune |
| 1173 | KY787088 | C | INDIA | 2016 | Pune |
| 1174 | KY787089 | C | INDIA | 2016 | Pune |
| 1175 | KY787090 | C | INDIA | 2016 | Pune |
| 1176 | KY787091 | C | INDIA | 2016 | Pune |
| 1177 | KY787092 | C | INDIA | 2016 | Pune |
| 1178 | KY787093 | C | INDIA | 2016 | Pune |
| 1179 | KY787094 | C | INDIA | 2016 | Pune |
| 1180 | KY787095 | C | INDIA | 2016 | Pune |
| 1181 | KY787096 | C | INDIA | 2016 | Pune |
| 1182 | KY787097 | C | INDIA | 2016 | Pune |
| 1183 | KY787098 | C | INDIA | 2016 | Pune |
| 1184 | KY787099 | C | INDIA | 2016 | Pune |
| 1185 | KY787100 | C | INDIA | 2016 | Pune |
| 1186 | KY787101 | C | INDIA | 2016 | Pune |
| 1187 | KY787102 | C | INDIA | 2016 | Pune |
| 1188 | KY787103 | C | INDIA | 2016 | Pune |
| 1189 | KY787104 | C | INDIA | 2016 | Pune |
| 1190 | KY787105 | C | INDIA | 2016 | Pune |
| 1191 | KY787106 | C | INDIA | 2016 | Pune |
| 1192 | KY787107 | C | INDIA | 2016 | Pune |
| 1193 | MG788713 | C | INDIA | 2016 | AGRA REGION |
| 1194 | MG788728 | C | INDIA | 2016 | AGRA REGION |
| 1195 | MG788738 | C | INDIA | 2016 | AGRA REGION |
| 1196 | MG788748 | C | INDIA | 2016 | AGRA REGION |
| 1197 | MH503757 | C | INDIA | 2016 | AGRA REGION |
| 1198 | MH503758 | C | INDIA | 2016 | AGRA REGION |
| 1199 | MH503759 | C | INDIA | 2016 | AGRA REGION |
| 1200 | MH503760 | C | INDIA | 2016 | AGRA REGION |
| 1201 | MH503763 | C | INDIA | 2016 | AGRA REGION |
| 1202 | MH503764 | C | INDIA | 2016 | AGRA REGION |
| 1203 | MH503765 | C | INDIA | 2016 | AGRA REGION |
| 1204 | MK331893 | C | INDIA | 2016 | Not Available |
| 1205 | MK331910 | C | INDIA | 2016 | Not Available |
| 1206 | MK331911 | C | INDIA | 2016 | Not Available |
| 1207 | MK331916 | C | INDIA | 2016 | Not Available |
| 1208 | MK331919 | C | INDIA | 2016 | Not Available |
| 1209 | MK331922 | C | INDIA | 2016 | Not Available |
| 1210 | MK331923 | C | INDIA | 2016 | Not Available |
| 1211 | MW118472 | C | INDIA | 2017 | Delhi |
| 1212 | MW118476 | C | INDIA | 2017 | Secunderabad |
| 1213 | MW118477 | C | INDIA | 2017 | Secunderabad |
| 1214 | MW118478 | C | INDIA | 2017 | Secunderabad |
| 1215 | MW118479 | C | INDIA | 2017 | Secunderabad |
| 1216 | MW118480 | C | INDIA | 2017 | Secunderabad |
| 1217 | MW118481 | C | INDIA | 2017 | Secunderabad |
| 1218 | MW118482 | C | INDIA | 2017 | Secunderabad |
| 1219 | MW118483 | C | INDIA | 2017 | Secunderabad |
| 1220 | MW118462 | C | INDIA | 2018 | Hooghly |
| 1221 | MW118463 | C | INDIA | 2018 | Hooghly |
| 1222 | MW118464 | C | INDIA | 2018 | Hooghly |
| 1223 | MW118465 | C | INDIA | 2018 | Hooghly |
| 1224 | MW118466 | C | INDIA | 2018 | Hooghly |
| 1225 | MW118467 | C | INDIA | 2018 | Jalandhar |
| 1226 | MW118468 | C | INDIA | 2018 | Jalandhar |
| 1227 | MW118469 | C | INDIA | 2018 | Jalandhar |
| 1228 | MW118470 | C | INDIA | 2018 | Jalandhar |
| 1229 | MW118471 | C | INDIA | 2018 | Delhi |
| 1230 | MW118473 | C | INDIA | 2018 | Pune |
| 1231 | MW118474 | C | INDIA | 2018 | Pune |
| 1232 | MW118475 | C | INDIA | 2018 | Pune |
| 1233 | MW118484 | C | INDIA | 2018 | Secunderabad |
| 1234 | MW118485 | C | INDIA | 2018 | Secunderabad |
| 1235 | MW118486 | C | INDIA | 2018 | Secunderabad |
| 1236 | MW118487 | C | INDIA | 2018 | Hyderabad |
| 1237 | MW118488 | C | INDIA | 2018 | Hyderabad |
| 1238 | MW118489 | C | INDIA | 2018 | Vadodara |
| 1239 | AY746389 | C | INDIA | Not Available | Pune |
| 1240 | EF694032 | C | INDIA | Not Available | Pune |
| 1241 | EF694034 | C | INDIA | Not Available | Pune |
| 1242 | EU429990 | C | INDIA | Not Available | Tamilnadu, Chennai |
| 1243 | KC016201 | C | INDIA | Not Available | Not Available |
| 1244 | KC016216 | C | INDIA | Not Available | Not Available |
| 1245 | KC016217 | C | INDIA | Not Available | Not Available |
| 1246 | KC016218 | C | INDIA | Not Available | Not Available |
| 1247 | KC016220 | C | INDIA | Not Available | Not Available |
| 1248 | KC016221 | C | INDIA | Not Available | Not Available |
| 1249 | KC016222 | C | INDIA | Not Available | Not Available |
| 1250 | KC016223 | C | INDIA | Not Available | Not Available |
| 1251 | KC016224 | C | INDIA | Not Available | Not Available |
| 1252 | KC016234 | C | INDIA | Not Available | Not Available |
| 1253 | KC016247 | C | INDIA | Not Available | Not Available |
| 1254 | KC016248 | C | INDIA | Not Available | Not Available |
| 1255 | KC016249 | C | INDIA | Not Available | Not Available |
| 1256 | KC016250 | C | INDIA | Not Available | Not Available |
| 1257 | KC016251 | C | INDIA | Not Available | Not Available |
| 1258 | KC016252 | C | INDIA | Not Available | Not Available |
| 1259 | KC016253 | C | INDIA | Not Available | Not Available |
| 1260 | KC016254 | C | INDIA | Not Available | Not Available |
| 1261 | KC016255 | C | INDIA | Not Available | Not Available |
| 1262 | KC016256 | C | INDIA | Not Available | Not Available |
| 1263 | KC016257 | C | INDIA | Not Available | Not Available |
| 1264 | KC016258 | C | INDIA | Not Available | Not Available |
| 1265 | KC016259 | C | INDIA | Not Available | Not Available |
| 1266 | KC016260 | C | INDIA | Not Available | Not Available |
| 1267 | KC016261 | C | INDIA | Not Available | Not Available |
| 1268 | KC016262 | C | INDIA | Not Available | Not Available |
| 1269 | KC016263 | C | INDIA | Not Available | Not Available |
| 1270 | KC016264 | C | INDIA | Not Available | Not Available |
| 1271 | KC016265 | C | INDIA | Not Available | Not Available |
| 1272 | KC016266 | C | INDIA | Not Available | Not Available |
| 1273 | KC016267 | C | INDIA | Not Available | Not Available |
| 1274 | KC016268 | C | INDIA | Not Available | Not Available |
| 1275 | KC016269 | C | INDIA | Not Available | Not Available |
| 1276 | KC016270 | C | INDIA | Not Available | Not Available |
| 1277 | KC016271 | C | INDIA | Not Available | Not Available |
| 1278 | KC016273 | C | INDIA | Not Available | Not Available |
| 1279 | KC016274 | C | INDIA | Not Available | Not Available |
| 1280 | KC016276 | C | INDIA | Not Available | Not Available |
| 1281 | KC016277 | C | INDIA | Not Available | Not Available |
| 1282 | KC016278 | C | INDIA | Not Available | Not Available |
| 1283 | KC016279 | C | INDIA | Not Available | Not Available |
| 1284 | KP227524 | C | INDIA | Not Available | Vellore |
| 1285 | KP227525 | C | INDIA | Not Available | Vellore |
| 1286 | KP227526 | C | INDIA | Not Available | Vellore |
| 1287 | KP227527 | C | INDIA | Not Available | Vellore |
| 1288 | KP227528 | C | INDIA | Not Available | Vellore |
| 1289 | KP227529 | C | INDIA | Not Available | Vellore |
| 1290 | KP227530 | C | INDIA | Not Available | Vellore |
| 1291 | KP227531 | C | INDIA | Not Available | Vellore |
| 1292 | KP227532 | C | INDIA | Not Available | Vellore |
| 1293 | KP227533 | C | INDIA | Not Available | Vellore |
| 1294 | KP227534 | C | INDIA | Not Available | Vellore |
| 1295 | KP227535 | C | INDIA | Not Available | Vellore |
| 1296 | KP227536 | C | INDIA | Not Available | Vellore |
| 1297 | KP227537 | C | INDIA | Not Available | Vellore |
| 1298 | KP227538 | C | INDIA | Not Available | Vellore |
| 1299 | KP227539 | C | INDIA | Not Available | Vellore |
| 1300 | KP227540 | C | INDIA | Not Available | Vellore |
| 1301 | KP227541 | C | INDIA | Not Available | Vellore |
| 1302 | KP227542 | C | INDIA | Not Available | Vellore |
| 1303 | KP227543 | C | INDIA | Not Available | Vellore |
| 1304 | KP227544 | C | INDIA | Not Available | Vellore |
| 1305 | KP227545 | C | INDIA | Not Available | Vellore |
| 1306 | KP227546 | C | INDIA | Not Available | Vellore |
| 1307 | KP227547 | C | INDIA | Not Available | Vellore |
| 1308 | KP227548 | C | INDIA | Not Available | Vellore |
| 1309 | KP227549 | C | INDIA | Not Available | Vellore |
| 1310 | KP227550 | C | INDIA | Not Available | Vellore |
| 1311 | KP227551 | C | INDIA | Not Available | Vellore |
| 1312 | KP227552 | C | INDIA | Not Available | Vellore |
| 1313 | KP227553 | C | INDIA | Not Available | Vellore |
| 1314 | KP227554 | C | INDIA | Not Available | Vellore |
| 1315 | KP227555 | C | INDIA | Not Available | Vellore |
| 1316 | KP227556 | C | INDIA | Not Available | Vellore |
| 1317 | KP227557 | C | INDIA | Not Available | Vellore |
| 1318 | KP227558 | C | INDIA | Not Available | Vellore |
| 1319 | KP227559 | C | INDIA | Not Available | Vellore |
| 1320 | KP227560 | C | INDIA | Not Available | Vellore |
| 1321 | KP227561 | C | INDIA | Not Available | Vellore |
| 1322 | KP227562 | C | INDIA | Not Available | Vellore |
| 1323 | KP227563 | C | INDIA | Not Available | Vellore |
| 1324 | KP227564 | C | INDIA | Not Available | Vellore |
| 1325 | KP227565 | C | INDIA | Not Available | Vellore |
| 1326 | KP227566 | C | INDIA | Not Available | Vellore |
| 1327 | KP227567 | C | INDIA | Not Available | Vellore |
| 1328 | KP227568 | C | INDIA | Not Available | Vellore |
| 1329 | KP227569 | C | INDIA | Not Available | Vellore |
| 1330 | KP227570 | C | INDIA | Not Available | Vellore |
| 1331 | KP227571 | C | INDIA | Not Available | Vellore |
| 1332 | KP227572 | C | INDIA | Not Available | Vellore |
| 1333 | KP227573 | C | INDIA | Not Available | Vellore |
| 1334 | KP227574 | C | INDIA | Not Available | Vellore |
| 1335 | KP227575 | C | INDIA | Not Available | Vellore |
| 1336 | KP227576 | C | INDIA | Not Available | Vellore |
| 1337 | KP227577 | C | INDIA | Not Available | Vellore |
| 1338 | KP227578 | C | INDIA | Not Available | Vellore |
| 1339 | KP227579 | C | INDIA | Not Available | Vellore |
| 1340 | KP227580 | C | INDIA | Not Available | Vellore |
| 1341 | KP227581 | C | INDIA | Not Available | Vellore |
| 1342 | KP227582 | C | INDIA | Not Available | Vellore |
| 1343 | KP227583 | C | INDIA | Not Available | Vellore |
| 1344 | KP227584 | C | INDIA | Not Available | Vellore |
| 1345 | KP227585 | C | INDIA | Not Available | Vellore |
| 1346 | KP227586 | C | INDIA | Not Available | Vellore |
| 1347 | KP227587 | C | INDIA | Not Available | Vellore |
| 1348 | KP227588 | C | INDIA | Not Available | Vellore |
| 1349 | KP227589 | C | INDIA | Not Available | Vellore |
| 1350 | KP227590 | C | INDIA | Not Available | Vellore |
| 1351 | KP227591 | C | INDIA | Not Available | Vellore |
| 1352 | KP227592 | C | INDIA | Not Available | Vellore |
| 1353 | KP227593 | C | INDIA | Not Available | Vellore |
| 1354 | KP227594 | C | INDIA | Not Available | Vellore |
| 1355 | KP227595 | C | INDIA | Not Available | Vellore |
| 1356 | KP227596 | C | INDIA | Not Available | Vellore |
| 1357 | KP227597 | C | INDIA | Not Available | Vellore |
| 1358 | KP227598 | C | INDIA | Not Available | Vellore |
| 1359 | KP227599 | C | INDIA | Not Available | Vellore |
| 1360 | KP227600 | C | INDIA | Not Available | Vellore |
| 1361 | KP227601 | C | INDIA | Not Available | Vellore |
| 1362 | KP227602 | C | INDIA | Not Available | Vellore |
| 1363 | KP227603 | C | INDIA | Not Available | Vellore |
| 1364 | KP227604 | C | INDIA | Not Available | Vellore |
| 1365 | KP227605 | C | INDIA | Not Available | Vellore |
| 1366 | KP227606 | C | INDIA | Not Available | Vellore |
| 1367 | KP227607 | C | INDIA | Not Available | Vellore |
| 1368 | KP227608 | C | INDIA | Not Available | Vellore |
| 1369 | KP227609 | C | INDIA | Not Available | Vellore |
| 1370 | KP227610 | C | INDIA | Not Available | Vellore |
| 1371 | KP227611 | C | INDIA | Not Available | Vellore |
| 1372 | KP227612 | C | INDIA | Not Available | Vellore |
| 1373 | KP227613 | C | INDIA | Not Available | Vellore |
| 1374 | KP227614 | C | INDIA | Not Available | Vellore |
| 1375 | KP227615 | C | INDIA | Not Available | Vellore |
| 1376 | KP227616 | C | INDIA | Not Available | Vellore |
| 1377 | KP227617 | C | INDIA | Not Available | Vellore |
| 1378 | KP227618 | C | INDIA | Not Available | Vellore |
| 1379 | KP227619 | C | INDIA | Not Available | Vellore |
| 1380 | KP227620 | C | INDIA | Not Available | Vellore |
| 1381 | KP227621 | C | INDIA | Not Available | Vellore |
| 1382 | KP227622 | C | INDIA | Not Available | Vellore |
| 1383 | KP227623 | C | INDIA | Not Available | Vellore |
| 1384 | KP227624 | C | INDIA | Not Available | Vellore |
| 1385 | KP227625 | C | INDIA | Not Available | Vellore |
| 1386 | KP227626 | C | INDIA | Not Available | Vellore |
| 1387 | KP227627 | C | INDIA | Not Available | Vellore |
| 1388 | KP227628 | C | INDIA | Not Available | Vellore |
| 1389 | KP227629 | C | INDIA | Not Available | Vellore |
| 1390 | KP227630 | C | INDIA | Not Available | Vellore |
| 1391 | KP227631 | C | INDIA | Not Available | Vellore |
| 1392 | KP227632 | C | INDIA | Not Available | Vellore |
| 1393 | KP227633 | C | INDIA | Not Available | Vellore |
| 1394 | KP227634 | C | INDIA | Not Available | Vellore |
| 1395 | KP227635 | C | INDIA | Not Available | Vellore |
| 1396 | KP227636 | C | INDIA | Not Available | Vellore |
| 1397 | KP227637 | C | INDIA | Not Available | Vellore |
| 1398 | KP227638 | C | INDIA | Not Available | Vellore |
| 1399 | KP227639 | C | INDIA | Not Available | Vellore |
| 1400 | KP227640 | C | INDIA | Not Available | Vellore |
| 1401 | KP227641 | C | INDIA | Not Available | Vellore |
| 1402 | KP227642 | C | INDIA | Not Available | Vellore |
| 1403 | KP227643 | C | INDIA | Not Available | Vellore |
| 1404 | KP227644 | C | INDIA | Not Available | Vellore |
| 1405 | KP227645 | C | INDIA | Not Available | Vellore |
| 1406 | KP227646 | C | INDIA | Not Available | Vellore |
| 1407 | KP227647 | C | INDIA | Not Available | Vellore |
| 1408 | KP227648 | C | INDIA | Not Available | Vellore |
| 1409 | KP227649 | C | INDIA | Not Available | Vellore |
| 1410 | KP227650 | C | INDIA | Not Available | Vellore |

Supplementary Table 2 – Reference sequences used for env gene region ML tree

| No | Accession | Subtype | Country | Sampling Year | Sampling City |
| --- | --- | --- | --- | --- | --- |
| 1 | L07651 | C | INDIA | 1991 | Goa |
| 2 | L07653 | C | INDIA | 1991 | Bombay |
| 3 | L07654 | C | INDIA | 1991 | Bombay |
| 4 | L07655 | C | INDIA | 1991 | Bombay |
| 5 | U07098 | C | INDIA | 1991 | Bombay |
| 6 | U07101 | C | INDIA | 1991 | Not Available |
| 7 | U07103 | C | INDIA | 1991 | Not Available |
| 8 | AB023804 | C | INDIA | 1993 | Not Available |
| 9 | AF067154 | C | INDIA | 1993 | Pune |
| 10 | AF067157 | C | INDIA | 1993 | Pune |
| 11 | AF067158 | C | INDIA | 1993 | Pune |
| 12 | U29179 | C | INDIA | 1993 | Not Available |
| 13 | U29694 | C | INDIA | 1993 | Not Available |
| 14 | U29695 | C | INDIA | 1993 | Not Available |
| 15 | U29696 | C | INDIA | 1993 | Not Available |
| 16 | U29697 | C | INDIA | 1993 | Not Available |
| 17 | U29698 | C | INDIA | 1993 | Not Available |
| 18 | U31362 | C | INDIA | 1993 | Not Available |
| 19 | U31363 | C | INDIA | 1993 | Not Available |
| 20 | AF067159 | C | INDIA | 1994 | Pune |
| 21 | AF286223 | C | INDIA | 1994 | Pune |
| 22 | AY713414 | C | INDIA | 1994 | Not Available |
| 23 | AF067155 | C | INDIA | 1995 | Pune |
| 24 | AF101119 | C | INDIA | 1995 | Andhra Pradesh |
| 25 | DQ367245 | C | INDIA | 1995 | Not Available |
| 26 | DQ367246 | C | INDIA | 1995 | Not Available |
| 27 | DQ367257 | C | INDIA | 1995 | Not Available |
| 28 | DQ367258 | C | INDIA | 1995 | Not Available |
| 29 | DQ367248 | C | INDIA | 1996 | Not Available |
| 30 | DQ367252 | C | INDIA | 1996 | Not Available |
| 31 | DQ367244 | C | INDIA | 1997 | Not Available |
| 32 | DQ367260 | C | INDIA | 1997 | Not Available |
| 33 | AF286231 | C | INDIA | 1998 | T'Nagar Madras |
| 34 | AF286232 | C | INDIA | 1998 | Churachandpur |
| 35 | AY049708 | C | INDIA | 1999 | GUJARAT |
| 36 | DQ367256 | C | INDIA | 1999 | Not Available |
| 37 | DQ367259 | C | INDIA | 1999 | Not Available |
| 38 | EF117268 | C | INDIA | 1999 | Not Available |
| 39 | EF117269 | C | INDIA | 1999 | Not Available |
| 40 | EF117270 | C | INDIA | 1999 | Not Available |
| 41 | EF117271 | C | INDIA | 1999 | Not Available |
| 42 | EF117272 | C | INDIA | 1999 | Not Available |
| 43 | EF117273 | C | INDIA | 1999 | Not Available |
| 44 | AY208967 | C | INDIA | 2000 | Pune |
| 45 | AY209192 | C | INDIA | 2000 | Pune |
| 46 | AY209193 | C | INDIA | 2000 | Pune |
| 47 | AY209194 | C | INDIA | 2000 | Pune |
| 48 | AY215067 | C | INDIA | 2000 | Pune |
| 49 | EF117265 | C | INDIA | 2000 | Not Available |
| 50 | EF117266 | C | INDIA | 2000 | Not Available |
| 51 | EF117267 | C | INDIA | 2000 | Not Available |
| 52 | EF117274 | C | INDIA | 2000 | Not Available |
| 53 | EU521729 | C | INDIA | 2000 | Pune |
| 54 | AY208968 | C | INDIA | 2001 | Pune |
| 55 | DQ367247 | C | INDIA | 2003 | Not Available |
| 56 | DQ367250 | C | INDIA | 2003 | Not Available |
| 57 | DQ367254 | C | INDIA | 2003 | Not Available |
| 58 | DQ381975 | C | INDIA | 2003 | Not Available |
| 59 | DQ381976 | C | INDIA | 2003 | Not Available |
| 60 | DQ381978 | C | INDIA | 2003 | Not Available |
| 61 | DQ381979 | C | INDIA | 2003 | Not Available |
| 62 | EF469243 | C | INDIA | 2003 | Bangalore |
| 63 | EU492868 | C | INDIA | 2003 | Not Available |
| 64 | EU492869 | C | INDIA | 2003 | Not Available |
| 65 | DQ367249 | C | INDIA | 2004 | Not Available |
| 66 | DQ367251 | C | INDIA | 2004 | Not Available |
| 67 | DQ367253 | C | INDIA | 2004 | Not Available |
| 68 | DQ367255 | C | INDIA | 2004 | Not Available |
| 69 | DQ381971 | C | INDIA | 2004 | Not Available |
| 70 | DQ381972 | C | INDIA | 2004 | Not Available |
| 71 | DQ381973 | C | INDIA | 2004 | Not Available |
| 72 | DQ381974 | C | INDIA | 2004 | Not Available |
| 73 | DQ381977 | C | INDIA | 2004 | Not Available |
| 74 | DQ381980 | C | INDIA | 2004 | Not Available |
| 75 | KF766537 | C | INDIA | 2004 | Tamilnadu |
| 76 | DQ325300 | C | INDIA | 2005 | Calcutta |
| 77 | DQ325301 | C | INDIA | 2005 | Calcutta |
| 78 | DQ325302 | C | INDIA | 2005 | Calcutta |
| 79 | DQ325303 | C | INDIA | 2005 | Calcutta |
| 80 | DQ325304 | C | INDIA | 2005 | Calcutta |
| 81 | DQ325305 | C | INDIA | 2005 | Calcutta |
| 82 | DQ325306 | C | INDIA | 2005 | Calcutta |
| 83 | DQ325307 | C | INDIA | 2005 | Calcutta |
| 84 | DQ325308 | C | INDIA | 2005 | Calcutta |
| 85 | DQ325309 | C | INDIA | 2005 | Calcutta |
| 86 | DQ325310 | C | INDIA | 2005 | Calcutta |
| 87 | DQ325311 | C | INDIA | 2005 | Calcutta |
| 88 | DQ325312 | C | INDIA | 2005 | Calcutta |
| 89 | DQ325313 | C | INDIA | 2005 | Calcutta |
| 90 | DQ325314 | C | INDIA | 2005 | Calcutta |
| 91 | DQ325315 | C | INDIA | 2005 | Calcutta |
| 92 | DQ325316 | C | INDIA | 2005 | Calcutta |
| 93 | DQ325317 | C | INDIA | 2005 | Calcutta |
| 94 | DQ325318 | C | INDIA | 2005 | Calcutta |
| 95 | DQ325319 | C | INDIA | 2005 | Calcutta |
| 96 | DQ325320 | C | INDIA | 2005 | Calcutta |
| 97 | DQ325321 | C | INDIA | 2005 | Calcutta |
| 98 | DQ325322 | C | INDIA | 2005 | Calcutta |
| 99 | DQ325323 | C | INDIA | 2005 | Calcutta |
| 100 | DQ325324 | C | INDIA | 2005 | Calcutta |
| 101 | DQ325325 | C | INDIA | 2005 | Calcutta |
| 102 | DQ325326 | C | INDIA | 2005 | Calcutta |
| 103 | DQ325327 | C | INDIA | 2005 | Calcutta |
| 104 | DQ325328 | C | INDIA | 2005 | Calcutta |
| 105 | DQ325329 | C | INDIA | 2005 | Calcutta |
| 106 | DQ325330 | C | INDIA | 2005 | Calcutta |
| 107 | DQ325331 | C | INDIA | 2005 | Calcutta |
| 108 | DQ325332 | C | INDIA | 2005 | Calcutta |
| 109 | DQ325334 | C | INDIA | 2005 | Calcutta |
| 110 | DQ325335 | C | INDIA | 2005 | Calcutta |
| 111 | DQ325336 | C | INDIA | 2005 | Calcutta |
| 112 | DQ325337 | C | INDIA | 2005 | Calcutta |
| 113 | DQ325338 | C | INDIA | 2005 | Calcutta |
| 114 | KF766540 | C | INDIA | 2005 | Tamilnadu |
| 115 | KF766541 | C | INDIA | 2005 | Tamilnadu |
| 116 | EU908214 | C | INDIA | 2006 | Not Available |
| 117 | EU908216 | C | INDIA | 2006 | Not Available |
| 118 | EU521727 | C | INDIA | 2007 | Pune |
| 119 | EU908218 | C | INDIA | 2007 | Not Available |
| 120 | EU908221 | C | INDIA | 2007 | Not Available |
| 121 | EU908222 | C | INDIA | 2007 | Not Available |
| 122 | JN400529 | C | INDIA | 2007 | Not Available |
| 123 | JN400538 | C | INDIA | 2007 | Not Available |
| 124 | EU908224 | C | INDIA | 2008 | Not Available |
| 125 | HQ897956 | C | INDIA | 2009 | Nagaland |
| 126 | HQ897957 | C | INDIA | 2009 | Nagaland |
| 127 | HQ897958 | C | INDIA | 2009 | Nagaland |
| 128 | HQ897959 | C | INDIA | 2009 | Nagaland |
| 129 | HQ897960 | C | INDIA | 2009 | Nagaland |
| 130 | HQ897962 | C | INDIA | 2009 | Nagaland |
| 131 | JF680935 | C | INDIA | 2009 | Not Available |
| 132 | JQ599356 | C | INDIA | 2011 | Not Available |
| 133 | JQ599357 | C | INDIA | 2011 | Not Available |
| 134 | KX069227 | C | INDIA | 2011 | Chennai |
| 135 | KX069228 | C | INDIA | 2011 | Chennai |
| 136 | KY929361 | C | INDIA | 2011 | Not Available |
| 137 | KY929362 | C | INDIA | 2011 | Not Available |
| 138 | KY929363 | C | INDIA | 2011 | Not Available |
| 139 | KY929364 | C | INDIA | 2011 | Not Available |
| 140 | KY929365 | C | INDIA | 2011 | Not Available |
| 141 | KY929366 | C | INDIA | 2011 | Not Available |
| 142 | KY929367 | C | INDIA | 2011 | Not Available |
| 143 | KY929368 | C | INDIA | 2011 | Not Available |
| 144 | MK521804 | C | INDIA | 2011 | Not Available |
| 145 | KY929369 | C | INDIA | 2012 | Not Available |
| 146 | KY929370 | C | INDIA | 2012 | Not Available |
| 147 | KX756602 | C | INDIA | 2013 | Not Available |
| 148 | KX756610 | C | INDIA | 2013 | Not Available |
| 149 | KX756611 | C | INDIA | 2013 | Not Available |
| 150 | KX756612 | C | INDIA | 2013 | Not Available |
| 151 | KX756613 | C | INDIA | 2013 | Not Available |
| 152 | KX756614 | C | INDIA | 2013 | Not Available |
| 153 | KX756615 | C | INDIA | 2013 | Not Available |
| 154 | KX069222 | C | INDIA | 2014 | Chennai |
| 155 | KX756600 | C | INDIA | 2014 | Not Available |
| 156 | KX756601 | C | INDIA | 2014 | Not Available |
| 157 | KX756603 | C | INDIA | 2014 | Not Available |
| 158 | KX756604 | C | INDIA | 2014 | Not Available |
| 159 | KX756605 | C | INDIA | 2014 | Not Available |
| 160 | KX756606 | C | INDIA | 2014 | Not Available |
| 161 | KX756607 | C | INDIA | 2014 | Not Available |
| 162 | KX756608 | C | INDIA | 2014 | Not Available |
| 163 | KX756609 | C | INDIA | 2014 | Not Available |
| 164 | MH899158 | C | INDIA | 2014 | Not Available |
| 165 | MZ397987 | C | INDIA | 2014 | Not Available |
| 166 | KX069219 | C | INDIA | 2015 | Chennai |
| 167 | KX069220 | C | INDIA | 2015 | Chennai |
| 168 | KX069221 | C | INDIA | 2015 | Chennai |
| 169 | KX069223 | C | INDIA | 2015 | Chennai |
| 170 | KX069224 | C | INDIA | 2015 | Chennai |
| 171 | KX069225 | C | INDIA | 2015 | Chennai |
| 172 | KX069226 | C | INDIA | 2015 | Chennai |
| 173 | KY713228 | C | INDIA | 2015 | Bengaluru |
| 174 | KY713229 | C | INDIA | 2015 | Bengaluru |
| 175 | KY713230 | C | INDIA | 2015 | Bengaluru |
| 176 | KY713231 | C | INDIA | 2015 | Bengaluru |
| 177 | KY713233 | C | INDIA | 2015 | Bengaluru |
| 178 | KY713234 | C | INDIA | 2015 | Bengaluru |
| 179 | KY713235 | C | INDIA | 2015 | Bengaluru |
| 180 | KY713236 | C | INDIA | 2015 | Bengaluru |
| 181 | KY713246 | C | INDIA | 2015 | Bengaluru |
| 182 | KY713248 | C | INDIA | 2015 | Bengaluru |
| 183 | MH899152 | C | INDIA | 2015 | Not Available |
| 184 | MN703367 | C | INDIA | 2016 | Not Available |
| 185 | MN703373 | C | INDIA | 2016 | Not Available |
| 186 | MN703343 | C | INDIA | 2017 | Not Available |
| 187 | MN703351 | C | INDIA | 2017 | Not Available |
| 188 | MN703377 | C | INDIA | 2017 | Not Available |
| 189 | MN703386 | C | INDIA | 2017 | Not Available |
| 190 | MT366192 | C | INDIA | 2017 | Not Available |
| 191 | MN275977 | C | INDIA | 2018 | Not Available |
| 192 | MN703360 | C | INDIA | 2018 | Not Available |
| 193 | MN703381 | C | INDIA | 2018 | Not Available |
| 194 | MN703391 | C | INDIA | 2018 | Not Available |
| 195 | MN703397 | C | INDIA | 2018 | Not Available |
| 196 | MN703402 | C | INDIA | 2018 | Not Available |
| 197 | AJ276221 | C | INDIA | Not Available | Calcutta |
| 198 | AJ276222 | C | INDIA | Not Available | CALCUTTA* |
| 199 | AJ291688 | C | INDIA | Not Available | Calcutta |
| 200 | AJ291689 | C | INDIA | Not Available | Calcutta |
| 201 | AJ291690 | C | INDIA | Not Available | Calcutta |
| 202 | AJ291691 | C | INDIA | Not Available | Calcutta |
| 203 | AJ292011 | C | INDIA | Not Available | CALCUTTA |
| 204 | AJ292012 | C | INDIA | Not Available | CALCUTTA |
| 205 | AJ292013 | C | INDIA | Not Available | CALCUTTA |
| 206 | AY653043 | C | INDIA | Not Available | Manipur |
| 207 | AY653044 | C | INDIA | Not Available | Manipur |
| 208 | AY653045 | C | INDIA | Not Available | Manipur |
| 209 | AY653046 | C | INDIA | Not Available | Manipur |
| 210 | AY653047 | C | INDIA | Not Available | Manipur |
| 211 | AY653048 | C | INDIA | Not Available | Manipur |
| 212 | AY653049 | C | INDIA | Not Available | Manipur |
| 213 | AY653050 | C | INDIA | Not Available | Manipur |
| 214 | AY653051 | C | INDIA | Not Available | Manipur |
| 215 | AY653052 | C | INDIA | Not Available | Manipur |
| 216 | AY653054 | C | INDIA | Not Available | Manipur |
| 217 | AY653055 | C | INDIA | Not Available | Manipur |
| 218 | AY653056 | C | INDIA | Not Available | Manipur |
| 219 | AY653057 | C | INDIA | Not Available | Manipur |
| 220 | AY653058 | C | INDIA | Not Available | Manipur |
| 221 | AY653059 | C | INDIA | Not Available | Manipur |
| 222 | AY653060 | C | INDIA | Not Available | Manipur |
| 223 | AY653061 | C | INDIA | Not Available | Manipur |
| 224 | AY653062 | C | INDIA | Not Available | Manipur |
| 225 | AY653063 | C | INDIA | Not Available | Manipur |
| 226 | AY653064 | C | INDIA | Not Available | Manipur |
| 227 | AY653065 | C | INDIA | Not Available | Manipur |
| 228 | AY653066 | C | INDIA | Not Available | Manipur |
| 229 | AY653067 | C | INDIA | Not Available | Manipur |
| 230 | AY653068 | C | INDIA | Not Available | Manipur |
| 231 | AY653071 | C | INDIA | Not Available | Manipur |
| 232 | AY775283 | C | INDIA | Not Available | Not Available |
| 233 | DQ149139 | C | INDIA | Not Available | Darjeeling |
| 234 | DQ149140 | C | INDIA | Not Available | Darjeeling |
| 235 | DQ149141 | C | INDIA | Not Available | Darjeeling |
| 236 | DQ149142 | C | INDIA | Not Available | Darjeeling |
| 237 | DQ149143 | C | INDIA | Not Available | Darjeeling |
| 238 | DQ149144 | C | INDIA | Not Available | Darjeeling |
| 239 | DQ149145 | C | INDIA | Not Available | Darjeeling |
| 240 | DQ149146 | C | INDIA | Not Available | Darjeeling |
| 241 | DQ149147 | C | INDIA | Not Available | Darjeeling |
| 242 | DQ149148 | C | INDIA | Not Available | Darjeeling |
| 243 | DQ149149 | C | INDIA | Not Available | Darjeeling |
| 244 | DQ149150 | C | INDIA | Not Available | Darjeeling |
| 245 | DQ149151 | C | INDIA | Not Available | Darjeeling |
| 246 | DQ149152 | C | INDIA | Not Available | Darjeeling |
| 247 | DQ398880 | C | INDIA | Not Available | Not Available |
| 248 | DQ398881 | C | INDIA | Not Available | Not Available |
| 249 | EF694032 | C | INDIA | Not Available | Pune |
| 250 | EF694034 | C | INDIA | Not Available | Pune |
| 251 | EU492870 | C | INDIA | Not Available | Not Available |
| 252 | EU492871 | C | INDIA | Not Available | Not Available |
| 253 | EU521728 | C | INDIA | Not Available | Pune |
| 254 | EU526660 | C | INDIA | Not Available | Nagaland |
| 255 | EU526661 | C | INDIA | Not Available | Nagaland |
| 256 | EU526663 | C | INDIA | Not Available | Nagaland |
| 257 | EU526664 | C | INDIA | Not Available | Nagaland |
| 258 | EU622000 | C | INDIA | Not Available | Pune |
| 259 | EU622001 | C | INDIA | Not Available | Pune |
| 260 | EU622004 | C | INDIA | Not Available | Pune |
| 261 | EU622006 | C | INDIA | Not Available | Pune |
| 262 | EU622009 | C | INDIA | Not Available | Pune |
| 263 | EU622012 | C | INDIA | Not Available | Pune |
| 264 | EU622015 | C | INDIA | Not Available | Pune |
| 265 | KP883221 | C | INDIA | Not Available | Not Available |
| 266 | KP883222 | C | INDIA | Not Available | Not Available |
| 267 | KP883223 | C | INDIA | Not Available | Not Available |
| 268 | KP883224 | C | INDIA | Not Available | Not Available |
| 269 | KP883225 | C | INDIA | Not Available | Not Available |
| 270 | KP883226 | C | INDIA | Not Available | Not Available |
| 271 | KP883227 | C | INDIA | Not Available | Not Available |
| 272 | KP883228 | C | INDIA | Not Available | Not Available |
| 273 | KP883229 | C | INDIA | Not Available | Not Available |
| 274 | KP883230 | C | INDIA | Not Available | Not Available |
| 275 | KP883231 | C | INDIA | Not Available | Not Available |
| 276 | KP883232 | C | INDIA | Not Available | Not Available |
| 277 | KP883233 | C | INDIA | Not Available | Not Available |
| 278 | KP883234 | C | INDIA | Not Available | Not Available |
| 279 | KP883236 | C | INDIA | Not Available | Not Available |
| 280 | KP883237 | C | INDIA | Not Available | Not Available |
| 281 | KP883238 | C | INDIA | Not Available | Not Available |
| 282 | KP883242 | C | INDIA | Not Available | Not Available |
| 283 | KP883245 | C | INDIA | Not Available | Not Available |
| 284 | U07100 | C | INDIA | Not Available | Not Available |
| 285 | U07102 | C | INDIA | Not Available | Not Available |

Supplementary Table 3 – Unnao HIV-1 sequences used for study.

| **No** | **Name** | **Pol gene Accession** | **Env gene**  **Accession** |
| --- | --- | --- | --- |
| 1 | NARI_18-5450_010721 | MW016009 | MW016023 |
| 2 | NARI_18-5451_010711 | MW016010 | MW016024 |
| 3 | NARI_18-5456_010051 | MW016011 | MW016025 |
| 4 | NARI_18-5464_010401 | MW016012 | MW016026 |
| 5 | NARI_18-5471_020821 | MW016013 | NA |
| 6 | NARI_18-5472_030831 | MW016014 | MW016027 |
| 7 | NARI_18-5473_010841 | MW016015 | MW016028 |
| 8 | NARI_18-5474_020851 | MW016016 | MW016029 |
| 9 | NARI_18-5476_010871 | MW016017 | NA |
| 10 | NARI_18-5478_010891 | MW016018 | MW016031 |
| 11 | NARI_18-5479_010901 | MW016019 | MW016032 |
| 12 | NARI_18-5480_010911 | MW016020 | MW016033 |
| 13 | NARI_18-5481_31750 | MW016021 | NA |
| 14 | NARI_18-5482_011100 | MW016022 | MW016034 |

NA – Sequence not available
