# Supplementary figures and images for: Exploring the Evolutionary History and Phylodynamics of Human Immunodeficiency Virus Type 1 Outbreak From Unnao, India Using Phylogenetic Approach
Source: Front Microbiol. 2022 May 18;13:848250. doi: 10.3389/fmicb.2022.848250 (PMC9158528; doi:10.3389/fmicb.2022.848250)

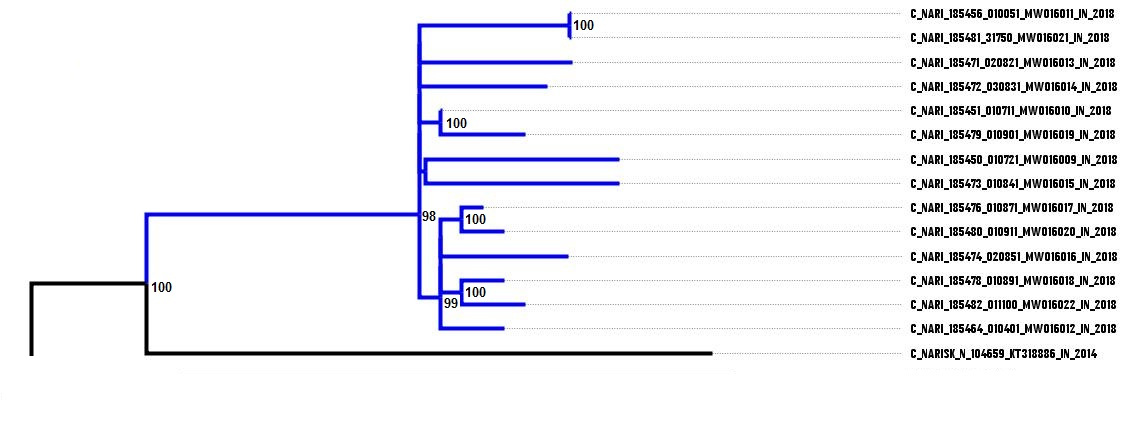

Supplement: Supplementary file 2 [file Image_1.JPEG]

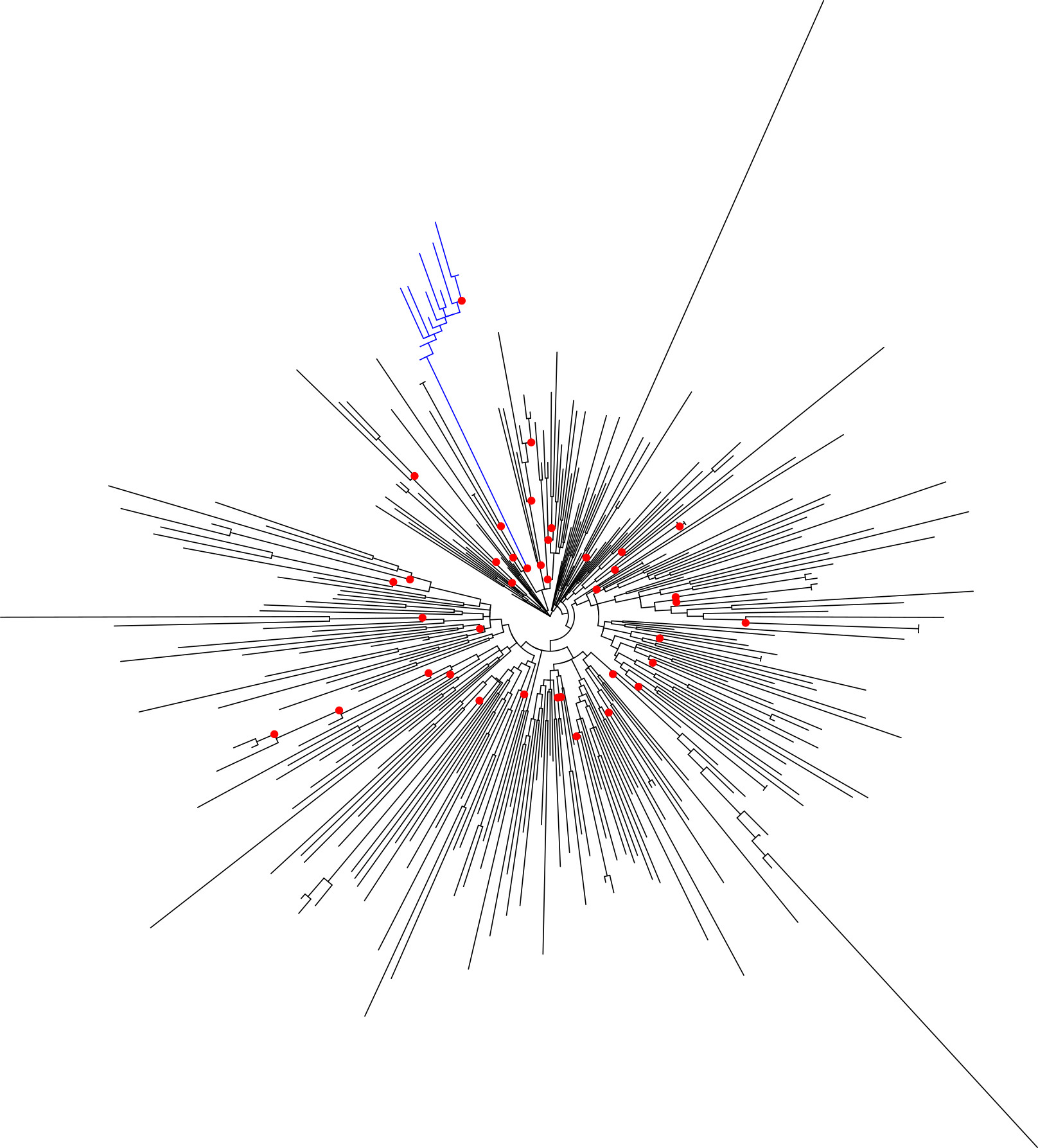

Supplement: Supplementary file 3 [file Image_2.JPEG]

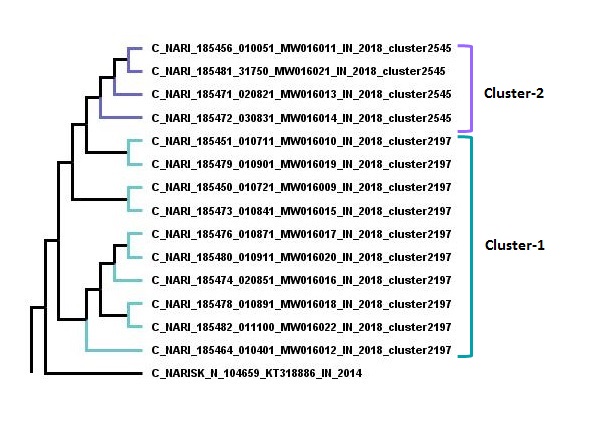

Supplement: Supplementary file 4 [file Image_3.JPEG]

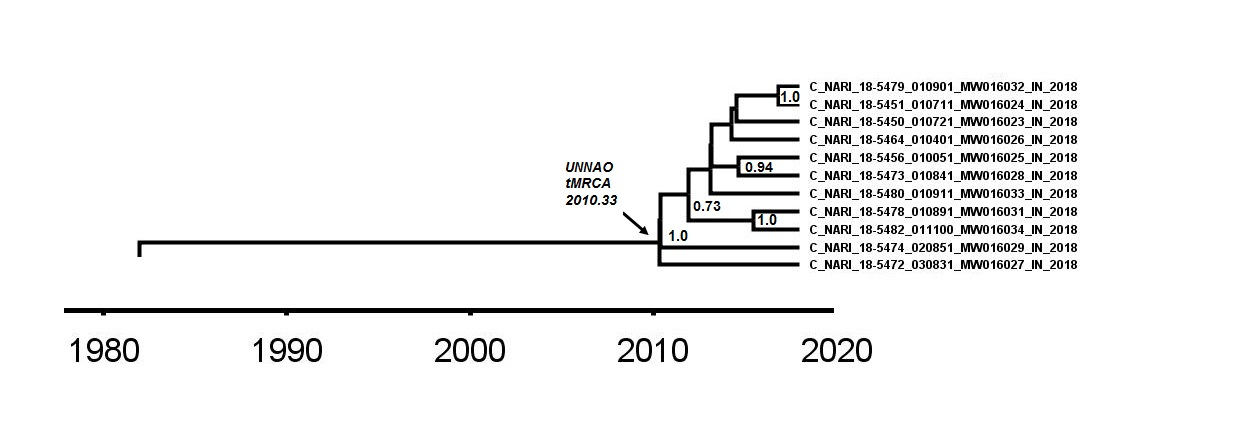

Supplement: Supplementary file 5 [file Image_4.JPEG]
